# Measuring Fisher Information Accurately in Correlated Neural Populations

**DOI:** 10.1371/journal.pcbi.1004218

**Published:** 2015-06-01

**Authors:** Ingmar Kanitscheider, Ruben Coen-Cagli, Adam Kohn, Alexandre Pouget

**Affiliations:** 1 Department of Basic Neuroscience, University of Geneva, Geneva, Switzerland; 2 Center of Learning and Memory and Department of Neuroscience, The University of Texas at Austin, Austin, Texas, United States of America; 3 Dominick Purpura Department of Neuroscience Albert Einstein College of Medicine, Bronx, New York, United States of America; 4 Department of Ophthalmology and Visual Sciences, Albert Einstein College of Medicine, Bronx, New York, United States of America; 5 Department of Brain and Cognitive Sciences, University of Rochester, Rochester, New York, United States of America; 6 Gatsby Computational Neuroscience Unit, London, United Kingdom; University of Tübingen and Max Planck Institute for Biologial Cybernetics, GERMANY

## Abstract

Neural responses are known to be variable. In order to understand how this neural variability constrains behavioral performance, we need to be able to measure the reliability with which a sensory stimulus is encoded in a given population. However, such measures are challenging for two reasons: First, they must take into account noise correlations which can have a large influence on reliability. Second, they need to be as efficient as possible, since the number of trials available in a set of neural recording is usually limited by experimental constraints. Traditionally, cross-validated decoding has been used as a reliability measure, but it only provides a lower bound on reliability and underestimates reliability substantially in small datasets. We show that, if the number of trials per condition is larger than the number of neurons, there is an alternative, direct estimate of reliability which consistently leads to smaller errors and is much faster to compute. The superior performance of the direct estimator is evident both for simulated data and for neuronal population recordings from macaque primary visual cortex. Furthermore we propose generalizations of the direct estimator which measure changes in stimulus encoding across conditions and the impact of correlations on encoding and decoding, typically denoted by *I_shuffle_* and *I_diag_* respectively.

## Author Summary

A central problem in systems neuroscience is to understand how the activity of neural populations is mapped onto behavior. Neural responses in sensory areas vary substantially upon repeated presentations of the same stimulus, and this limits the reliability with which two similar stimuli can be discriminated by any read-out of neural activity. Fisher information provides a quantitative measure of the reliability of the sensory representation, and it has been used extensively to analyze neural data. Traditional methods for quantifying Fisher information rely on decoding neural activity; however, optimizing a decoder requires larger amounts of data than available in typical experiments, and as a result decoding-based estimators systematically underestimate information. Here we introduce a novel estimator that can accurately determine information with far less data, and that runs orders of magnitude faster. The estimator is based on analytical calculation, and corrects the bias that arises when estimating information directly from limited data. The analytical guarantee of an unbiased estimator and its computational simplicity will allow experimentalists to compare coding reliability across behavioral conditions and monitor it over time.

## Introduction

The advent of technical advances like multi-electrode recordings and calcium imaging allows the simultaneous recording of an ever increasing number of neurons. The availability of this data allows us to explore not only the qualitative properties of the neural code but also the reliability of coding.

One challenge of assessing coding reliability from neural recordings is that the number of available trials is typically quite limited. In contrast to the downstream circuitry, which potentially had a lifetime of experience to learn the statistics of neural responses, an experimenter recording neural activity only has finite data both to fit the response statistics and to assess the coding reliability. This requires efficient methods to achieve an accurate estimate of reliability with as few trials as possible.

In the frequently considered case of population coding for a continuous stimulus variable, a common approach to quantifying coding reliability is to assess how well a decoder of population patterns of activity can discriminate between two slightly different stimulus values (e.g. [1–3]). The discrimination threshold (i.e. the smallest difference between two stimuli that can be correctly classified say 80% of the time) is determined by the variance of the decoder’s estimate for a fixed stimulus value. A well-known result of information theory, the Cramer-Rao bound, specifies that the optimal decoder variance is larger than or equal to the inverse of the Fisher information [4,5]. Therefore Fisher information quantifies the amount of information that can be extracted by the ideal observer (or, equivalently, an optimal decoder).

In this paper, we focus on estimating linear Fisher information—the information that can be extracted by the locally optimal linear estimator, i.e. a linear estimator optimized to the response statistics around a specific stimulus value [1]. Linear Fisher information is a lower bound on Fisher information, and captures the fraction of the total information contained in the trial-averaged responses which can be extracted without further non-linear processing. For example, a population of V1 neurons typically has substantial linear information about orientation, since its trial-averaged responses, the tuning curves, depend on orientation. V1 neurons also encode information about faces. This information, however, requires sophisticated non-linear processing to be extracted, and hence linear Fisher information will be low. For such a stimulus one would expect to measure higher linear Fisher information in higher visual areas than in V1 [6].

The linear Fisher information of a given population is determined by its tuning curves and covariance matrix (Materials and Methods, Section 1):

$$I=f\prime {\left(\theta \right)}^{T}\Sigma^{-1}f\prime (\theta ),$$ (1)

where **f**(*θ*) is the vector of tuning curves with entries *f*_*i*_(*θ*), *i* = 1.*N*, the prime denotes derivation with respect to the stimulus value *θ*, and ∑_*ij*_ is the noise covariance matrix [7,8]. However, accurately determining tuning curves and covariance matrices from finite data is difficult. As shown in [9], only the part of the covariance matrix proportional to **f**′(*θ*)**f**′(*θ*)^*T*^—termed differential correlations—limits Fisher information in large populations. Differential correlations can be extremely small and therefore small errors in estimating correlations can have a huge impact on information. As a result, measurement errors in **f**′ and Σ lead to large biases in the estimate of *I*.

There is at least one method for dealing with these biases. Moreno et al. [9] showed recently that a lower bound on linear Fisher information can be obtained with a cross-validated decoder even when differential correlations are present. In this approach (inspired by [1]), the data are split into a training set which is used to train a decoder, and a validation set which measures information by assessing the reliability of the decoder. As we show here, this method generally underestimates the true information, and will have a small bias only when the number of trials is much larger than the number of neurons. This is because a cross-validated decoder trained on finite data is typically suboptimal, resulting in a higher variance on the test set than the optimal decoder. This is a serious problem for experimental data, as the number of trials is rarely large enough to prevent biases with this approach.

In this paper, we show that for small number of trials, there is a better alternative to estimating Fisher information than decoding. The information can be estimated directly from Eq (1), based on the empirically measured tuning curves and covariance matrix. The key is to correct Fisher information for biases that are introduced by computing nonlinear functions of the tuning curves and covariance matrix estimated from limited data. We provide analytical expressions, and corrections, for the biases and show that the resulting bias-corrected estimator is much more accurate than the decoding method for a fixed number of trials while being much faster to compute. Furthermore, we provide a closed-form expression for the variance of the estimator. We illustrate the results on both synthetic data and data recorded in primate visual cortex.

Decoding methods are also often used to measure changes in the reliability of neural codes between conditions [10] and to assess information loss due to suboptimal readout [2,11,12]. We show that in these cases too, a bias-corrected direct estimate of information is often better than the ones obtained with cross-validated decoding. Likewise, this approach works well for estimating *I*_*shuffle*_, the information in a data set in which responses have been shuffled across trials, and *I*_*diag*_, the information recovered by using a factorized decoder, i.e., a decoder that ignores correlations.

## Results

**Fig 1:**
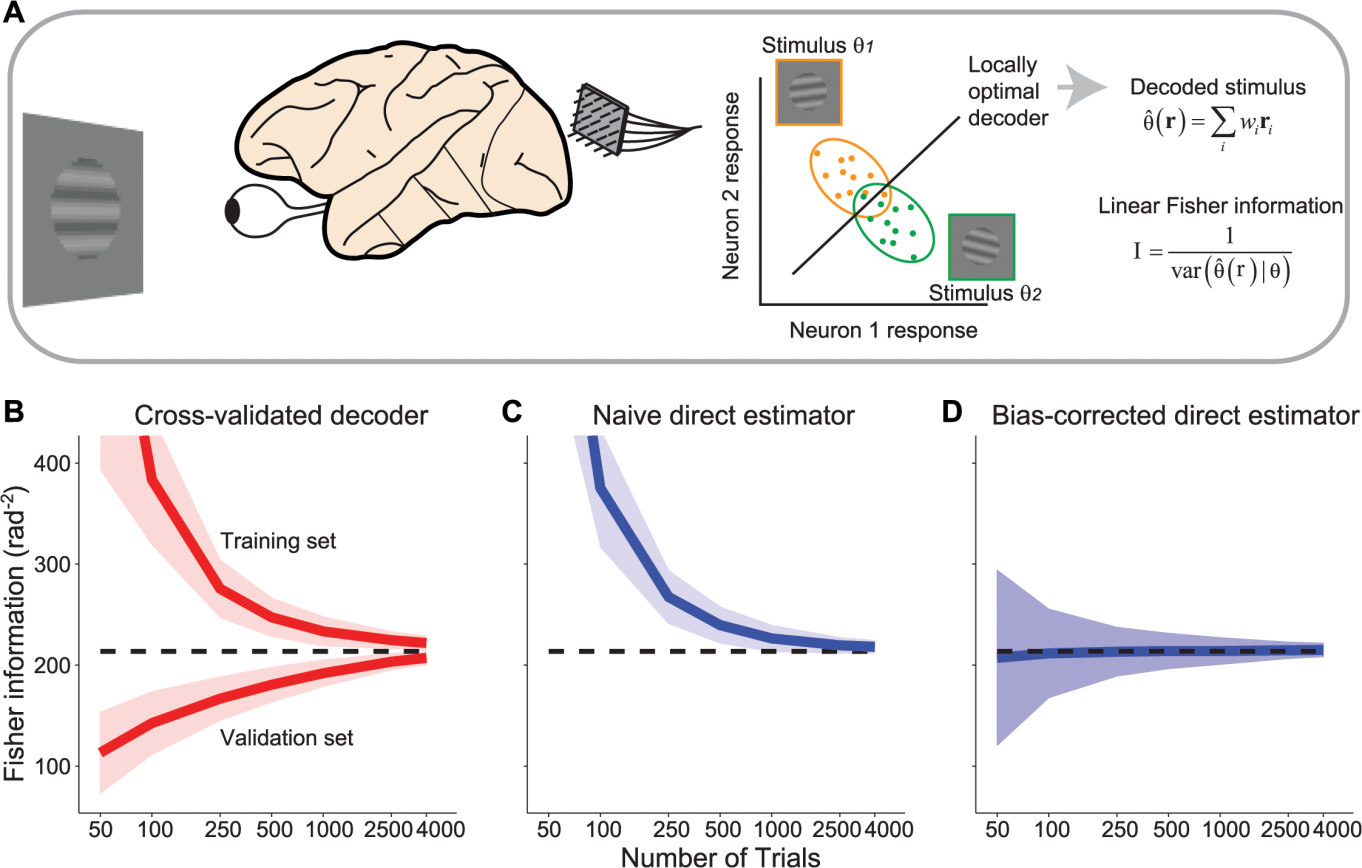
Stimulus decoding and Fisher information. (**a**) Left: In a typical experiment, the responses of a population of neurons are recorded simultaneously while a visual stimulus is presented. Middle: Population responses (*N* = 2 in the cartoon) to two different stimulus values (orange vs. green symbols) are collected over many trials. Right: The optimal decoding weights,*w*_*i*_, are applied to the population response, **r**, to obtain an estimate of the stimulus, $\hat{\theta }$. Linear Fisher information corresponds to the inverse of the variance of such estimates, across trials where the same stimulus was presented. (**b**) Ordinate: Fisher information in a population of *N* = 50 model neurons, estimated by a linear decoder as illustrated in (**a**), using cross-validation with early stopping. Abscissa: number of trials per stimulus condition. We ran 200 experiments for each trial. The top line is the information estimated from the training set; the bottom line is the information estimated from the validation set; the two will converge asymptotically. The dashed line is the true information value in the simulated population. The continuous lines represent the mean, the shaded area represents ±1 std across experiments, computed by bootstrap. (**c**) Fisher information obtained by directly estimating the tuning curves and covariance, and then applying Eq (11). The continuous lines represent the mean, the shaded area represents ±1 std across experiments, computed by bootstrap. (**d**) Similar to (**c**), but after correcting for the estimation bias, according to Eq (2). The continuous lines represent the mean, the shaded area represents ±1 std across experiments, computed analytically using Eq (19).

In a typical discrimination task the subject is asked to distinguish two similar stimuli, *θ* ^+^ = *θ* + *dθ* and *θ* ^−^ = *θ*−*dθ*. To measure Fisher information, a measure of discriminability, we consider neural responses to the two stimuli and estimate the performance of an optimal unbiased linear decoder to classify the stimulus. Fisher information is the inverse of the variance of the estimate of the stimulus based on this optimal linear decoder (see Fig 1A; details are provided in Materials and Methods Section 1).

### Summary of the model used for simulations

We first consider simulated responses from *N* neurons to the two discrimination stimuli, which are each repeated for *T* trials. Responses were generated using a model of orientation-selective units, such as those found in primary visual cortex. Inputs to the model were Gabor images corrupted by white pixel noise. Model neurons were represented by Gabor linear filters, whose output was half-rectified, and further corrupted by independent Poisson noise to produce realistic response variability. This is a doubly stochastic model, with part of the variability induced by image pixel noise, and part due to the Poisson step. Due to image noise and filter overlap, the network contains noise correlations which decay with tuning similarity, consistent with a wealth of experimental data [13]. We generated synthetic population responses from a network with *N* = 50 neurons (except where noted). Each simulated experiment comprised *T* trials per orientation. The number of trials varied between 50 and 4000, and for each *T* we ran 200 experiments. We computed the ground truth information for the model using the analytical expressions (Eq (1)) for the tuning curves and noise covariances. Further details about the model and simulations are provided in Materials and Methods Section 8.

### Measuring Fisher information with cross-validated decoding

Measuring Fisher information with cross-validated decoding requires splitting the data in a training and validation set. The training set is used to find a good estimate for the optimal decoder, while the validation set estimates its performance. Unless the number of trials available is much larger than the number of neurons, this method tends to overfit the training set, which in turn leads to severe underestimation of Fisher information for the validation set. A straightforward method to address this is early stopping. Early stopping splits the data in three sets, the training, test and validation set. It then proceeds by performing gradient descent on the training set while monitoring the error on the test set. As soon as the error in the test set starts increasing, the training is stopped. The Fisher information is then taken to be the inverse variance of the decoder’s estimates in the validation set (see Materials and Methods section 1, for details).

In Fig 1B we evaluate the performance of the decoder on artificial data obtained from the model described above. We see that cross-validated decoding using early stopping underestimates information (dashed line) substantially unless the number of trials is much larger than the number of neurons (e.g., by a factor of 25 to reach 90% of the true information; see Materials and Methods, Section 8). The reason is residual overfitting: for a small number of trials, the best-performing decoder in the training set will still be suboptimal and therefore underestimate information in the validation set.

### Measuring Fisher information using direct estimation

An estimate of Fisher information can be obtained directly from Eq (1), using the empirical covariance matrix and an estimate of the tuning curve derivative obtained from the difference in mean responses of the two presented stimuli. However, this naïve estimate of Fisher information substantially overestimates the true Fisher information (Fig 1C).

The reason for this overestimation is that the expression for Fisher information (Eq (1)) has a non-linear dependence both on the covariance matrix and on the tuning curve derivative, since the former is inverted and the latter is squared. Even though the empirical covariance matrix and difference in mean responses are unbiased estimators, the bias is reintroduced by the non-linear transformations. Consider for instance the simple case of a Gaussian variable *X* with true mean *μ* and variance *σ* ^2^. If we collect *T* measurements, *x*_1…*T*_, then the estimate of the mean $\hat{\mu }=\frac{1}{T}\sum_{i=1}^{T}x_{i}$ is unbiased, and has a variance $Var\;\hat{\mu }=\sigma^{2}/T$ (across several experiments, each with *T* measurements). Suppose now we are interested in estimating the square, *μ* ^2^. Then the naïve estimator, ${\hat{\mu }}^{2}$, is biased by the basic fact that $\langle {\hat{\mu }}^{2}\rangle =\mu^{2}+Var\;\hat{\mu }$. The bias vanishes if *T* is large enough, but can be substantial when based on limited measurements.

Fortunately it is possible to calculate the bias analytically and correct for it, if one assumes Gaussian response variability (but the results are robust to realistic violations of this assumption, as we illustrate below) and has more trials than neurons (more exactly, the empirical covariance matrix is invertible). In this case, the sampling distribution of the empirical covariance matrix is given by the Wishart distribution and its inverse by the inverse Wishart distribution. The bias of the inverse Wishart distribution is well-known [14]. The quadratic appearance of the tuning curve derivative introduces an additional bias. Once an analytical expression is derived for such biases, they can also be easily corrected (see Materials and Methods, Section 3 for full derivation), yielding an unbiased estimate of Fisher information:

$${\hat{I}}_{bc}={\frac{d\mu }{d\theta }}^{T}S^{-1}\frac{d\mu }{d\theta }\left(\frac{2T-N-3}{2T-2}\right)-\frac{2N}{Td\theta^{2}}$$ (2)

where **μ** and *S* represent the empirical mean and covariance, respectively.

The variance of the bias-corrected estimator can also be calculated analytically (Materials and Methods, Section 4, Eq (19); S1 Fig), and can be used to obtain error bars. The expression for the variance shows that it diverges if *T* = (*N* + 5) / 2; furthermore the empirical covariance matrix is invertible with probability 1 only if *T* > (*N* + 2) / 2, and therefore direct estimation cannot be used for smaller values of *T*.

**Fig 2:**
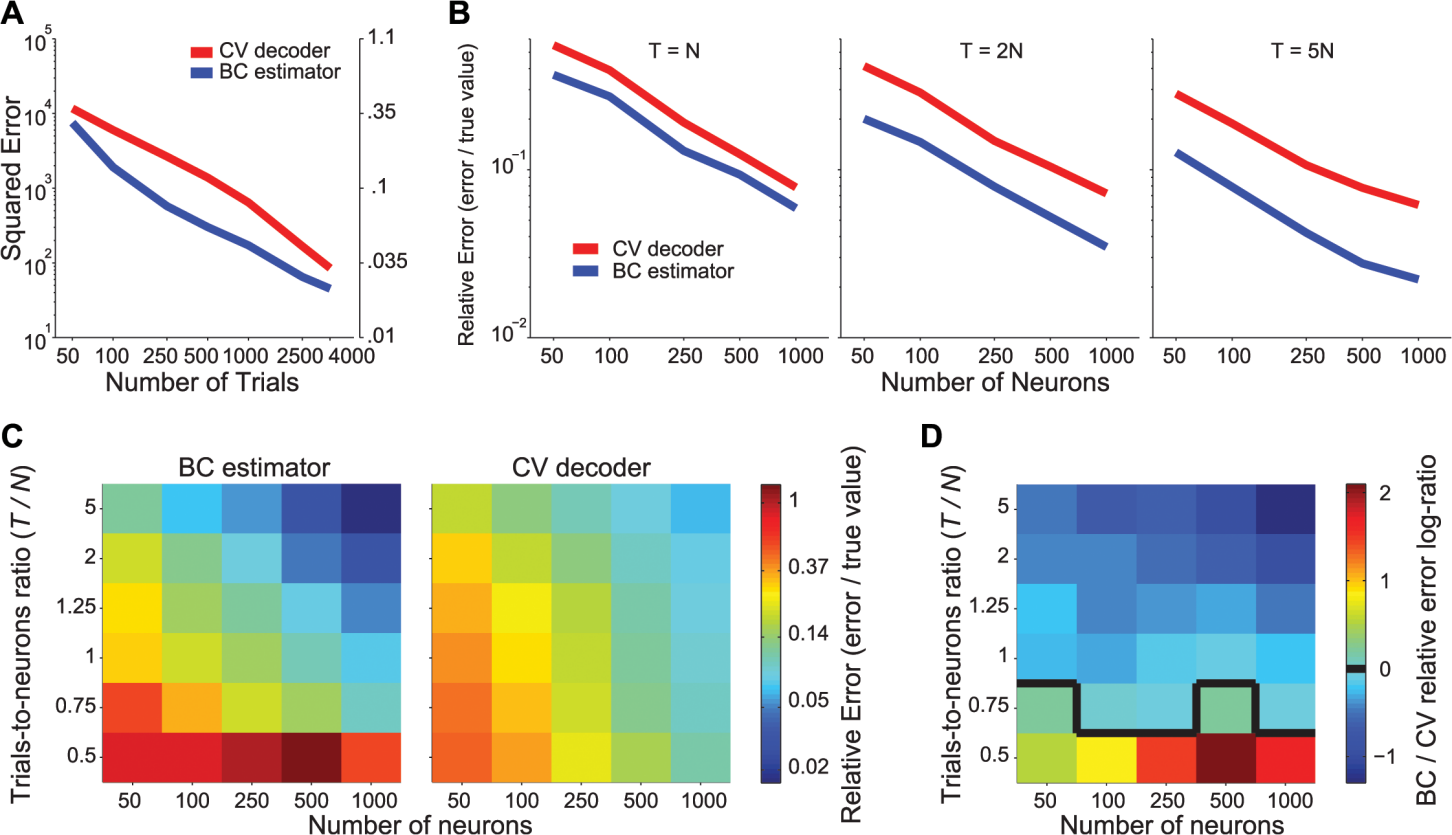
Comparison of the estimation errors of the decoder and the direct bias-corrected method. (**a**) Mean squared error (MSE) of the cross-validated decoder-based estimate (CV decoder, red) and the bias-corrected direct estimator (BC estimator, blue) calculated from the estimates of Fig 1 (*N* = 50 neurons). The ordinate axis on the right indicates the corresponding relative error, namely $\sqrt{\text{MSE}}/I$. (**b**) Relative error for CV decoder (red) and BC estimator (blue), for different population sizes (abscissa). The number of trials was set to 1, 2, or 5 times the number of neurons (indicated at the top of each panel). (**c**) Relative error for CV decoder (right) and BC estimator (left), for different population sizes (abscissa) and numbers of trials expressed as proportion of the number of neurons (ordinate). (**d**) Log-ratio of relative errors for BC vs. CV from panel (**c**). The black contour separates cases in which the CV estimator is more accurate than BC (warm colors) from cases in which BC is more accurate (cold colors).

The performance of the bias-corrected estimator can be seen in Fig 1D. It closely approximates the true information, even for small numbers of trials, and is on average unbiased. As expected, the variance of the estimate increases as the number of trials decreases. In Fig 2A, we compare the expected squared error (and the corresponding relative error, i.e. the ratio between estimation error and true value) of the cross-validated decoding method with the bias-corrected direct estimator. For all simulations illustrated (*T* > *N*), the direct estimator is more reliable than the decoding estimate. For instance, with 250 trials, the bias-corrected estimate is within 11% of the true information while the decoder estimate is within 24%. To achieve the same level of accuracy as the bias-corrected method for 250 trials, the decoder estimate requires 1000 trials. Fig 2B shows that the improvement of the bias-corrected estimator over decoding is roughly independent of the population size, and increases with the number of trials between *T* = *N* and *T* = 5 *N*. For a smaller number of trials, in the range (*N* + 5) / 2 < *T* < *N*, the bias-corrected estimator can be calculated but it may be less accurate than the decoding estimate (Fig 2C and 2D).

### Assessing context-dependent encoding and decoding

A broad class of experimental questions involves estimating the information that a decoder which is trained in one experimental condition can extract in a different experimental condition. An important example is whether changes in the sensory representation (i.e. the encoding stage) cause behavioral changes following experimental manipulations such as the allocation of attention [10,15], perceptual learning [16,17] or adaptation [18,19]. Another example is whether the representation of orientation in primary visual cortex is invariant to image contrast; that is whether a decoder specialized for one contrast level can extract all the information from population responses to another contrast level [2,3].

One way to test for context-dependent encoding and decoding is to train a decoder on data collected before the manipulation and then compare its performance on validation data collected before vs. after the manipulation. However, this approach leads to an underestimation of information, for the reasons discussed above. We propose instead a direct, unbiased estimator for the general case of a decoder trained on dataset A and tested on dataset B (analogous to the ‘unfaithful model’ discussed in [11]). The optimal decoding weights for dataset A are [20]

$$w_{A}\propto \Sigma_{A}^{-1}f{'}_{A},$$ (3)

The information that can be extracted by such decoder from dataset B is

$$I_{AB}=\frac{{\left(f{'}_{B}^{T}w_{A}\right)}^{2}}{w_{A}^{T}\Sigma_{B}w_{A}}=\frac{{\left(f{'}_{B}^{T}\Sigma_{A}^{-1}f{'}_{A}\right)}^{2}}{f{'}_{A}^{T}\Sigma_{A}^{-1}\Sigma_{B}\Sigma_{A}^{-1}f{'}_{A}}.$$ (4)

A bias-corrected estimator of the expression (4) is given in Materials and Methods, Section 6.

**Fig 3:**
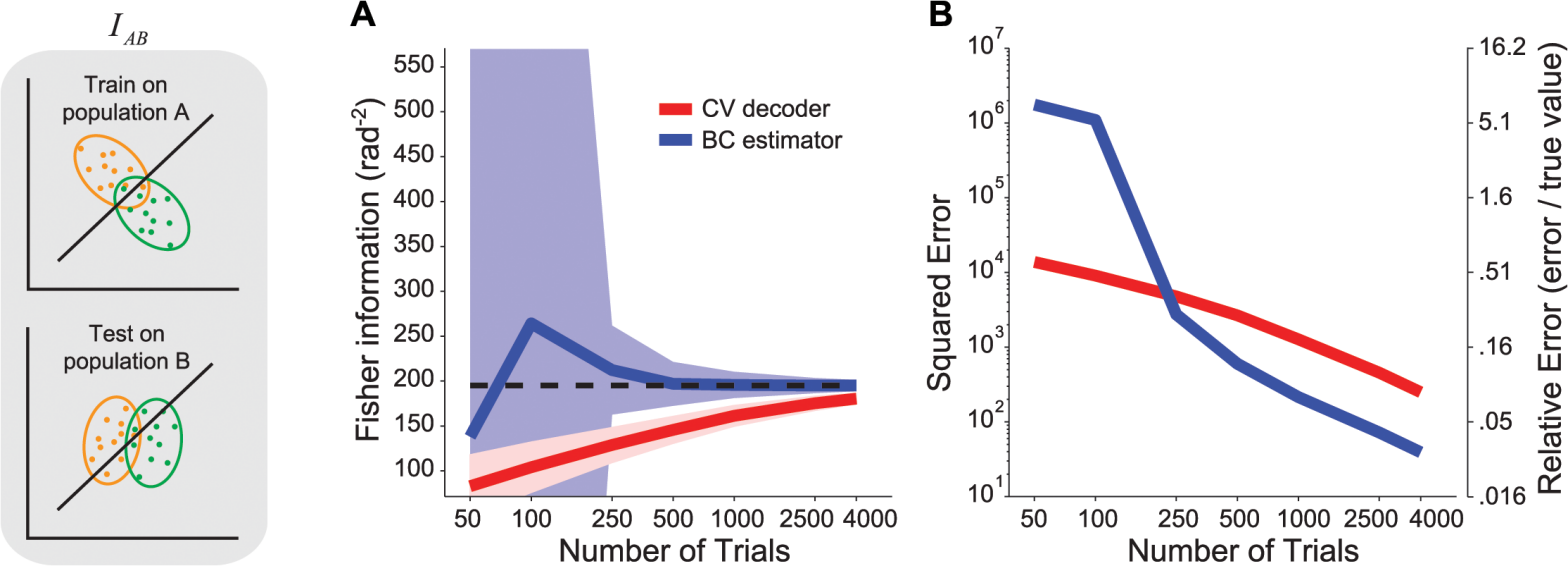
Fisher information estimates for the general case of a decoder trained and tested on different response statistics. Left: Illustration of the scenario. Decoding weights are optimized for data recorded from population A, then tested on data from population B. Note that the decision boundary derived from A is not optimal for B. (**a**) Estimate of the Fisher information obtained by decoding (red) or direct estimation with bias correction (blue). The continuous lines represent the mean, the shaded area represents ±1 std across experiments, computed by bootstrap. (**b**) MSE of the decoder-based estimate (red) and the direct estimator (blue).

We compared the cross-validated decoding method with the bias-corrected direct estimators on synthetic data. We considered two separate sets of covariance matrices and tuning curves, such that the optimal decoder was different for the two cases, and therefore the true crossed information *I*_*AB*_ was smaller than the true information in the second population, *I*_*B*_ (Materials and Methods, Section 7). We then generated data from those sets of tuning and covariances to evaluate the estimators. In Fig 3 we show that, for *N* = 50, as soon as *T* ≥ 5 *N*, the direct estimator is more reliable than the decoding estimate. Thus, in this case, similar to Fig 2D, there is still a cross-over in performance between the direct estimator and the decoder, but it happens at a larger number of trials than *N*. However, since we could not find a closed-form expression for the variance of *I*_*AB*_, it is hard to make a general statement about the precise point of cross-over, which will depend on the statistics of the datasets A and B.

### Directly measuring the impact of correlations on encoding and decoding

Sometimes experimental studies seek not only to estimate the information in a given population but also how that information is affected by correlations. Two widely used measures are *I*_*shuffle*_, which quantifies how information *encoding* is affected by correlations, i.e. how much information is present in a population with the same marginal statistics but no correlations; and *I*_*diag*_, which quantifies how information *decoding* is affected by correlations, i.e. how much information can be retrieved from the correlated population if the decoder does not model correlations [21,22]. *I*_*shuffle*_ is typically measured using cross-validated decoding after the neural responses are shuffled across trials. This shuffling destroys correlations and by comparing *I*_*shuffle*_ to the information in the original data, one can thus assess the impact of correlations on information encoding. To calculate *I*_*diag*_, a decoder is trained on shuffled data and tested on the original data, ensuring that the decoder cannot model correlations.

We propose to measure *I*_*shuffle*_ and *I*_*diag*_ directly using bias-corrected direct estimators. *I*_*shuffle*_ is defined as:

$$I_{shuffle}=f\prime^{T}\Sigma_{shuffle}^{-1}f\prime =\sum_{i}\frac{f_{i}\prime^{2}}{\sigma_{i}^{2}},$$ (5)

where $\sigma_{i}^{2}$ are the marginal variances of neural responses. An unbiased estimator of *I*_*shuffle*_ is given by

$${\hat{I}}_{bc,shuffle}=\sum_{i}\frac{{\left(d\mu_{i}/d\theta \right)}^{2}}{s_{i}^{2}}\frac{\left(T-2\right)}{\left(T-1\right)}-\frac{2N}{Td\theta^{2}}\;,$$ (6)

where $s_{i}^{2}$ is the unbiased estimator of the sample variance of neuron *i* (see Materials and Methods, Section 5). Note that Eq (6) does not require actually shuffling the data. This is yet another advantage of this technique over decoding approaches: shuffling cannot remove correlations entirely due to the finite number of trials, thus introducing an additional source of estimation error for decoder-based methods.

**Fig 4:**
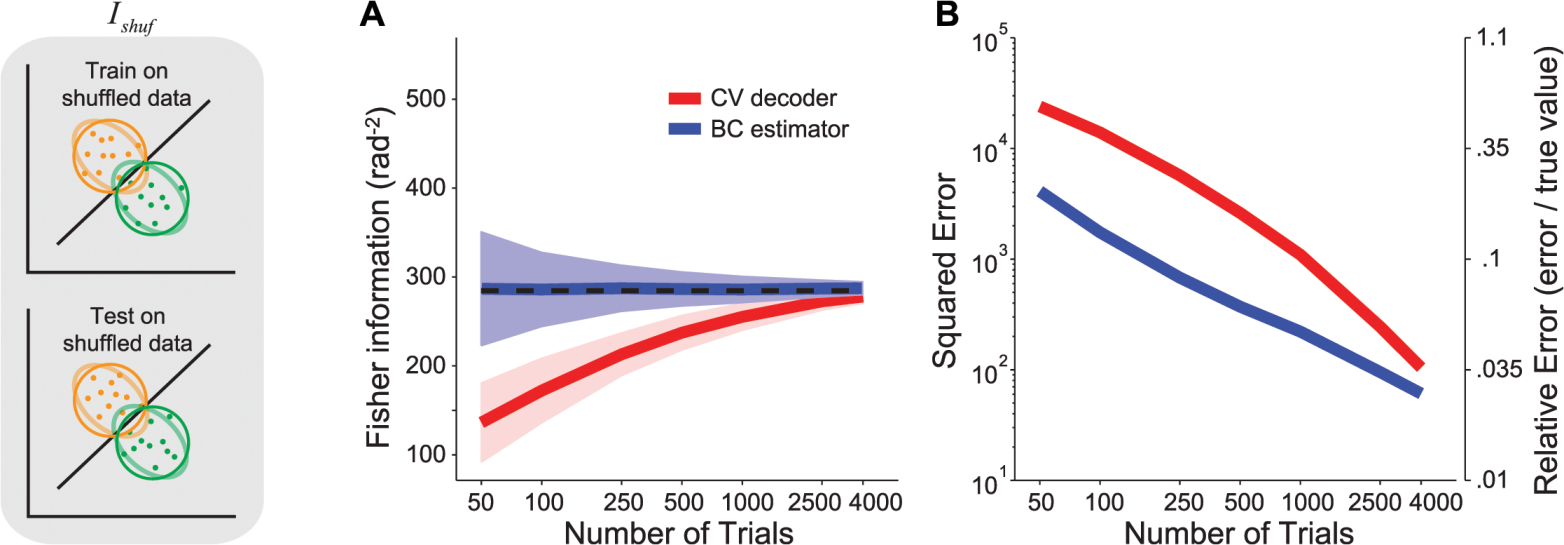
Fisher information estimates for an independent population. Left: Illustration of the scenario. The decoder is trained and tested on shuffled data, i.e. data where correlations have been destroyed by randomly permuting across trials the responses of each neuron independently. The faint lines represent the covariance ellipses of the original data, before shuffling. (**a**) Estimate of the Fisher information obtained by decoding (red) or direct estimation with bias correction (blue). The continuous lines represent the mean, the shaded area represents ±1 std across experiments, computed by bootstrap. (**b**) MSE of the decoder-based estimate (red), and the direct estimator (blue).

In Fig 4, we compare the cross-validated decoding method with the bias-corrected direct estimator for *I*_*shuffle*_. With *N* = 50 neurons, for *T* > *N*, the direct estimator is more reliable than the decoding estimate, and for small numbers of trials (*T* < 1000) the gap between the decoder and the direct estimator is even larger than in Fig 2A. This is because the decoder-based estimate of *I*_*shuffle*_ involves training on shuffled data, which can lead to large errors. Indeed, the decoding method works better when the code is highly redundant, which is to say, when the information saturates as the number of decoded neurons increases (or equivalently, when the code contains differential correlations [9]). Thanks to this redundancy, decoders that deviate slightly from the optimal (e.g. by placing too little weight on the most informative neurons) can still recover a large proportion of the information. Once the data are shuffled, the redundancies are gone. As a result, a small error in the decoder will lead to poor performance on the shuffled data [9]. Therefore, it is expected that a slightly suboptimal decoder (e.g. due to finite training set size) will miss a larger proportion of the information in the shuffled case than in the original case. Furthermore, this weakness of decoding methods becomes even more apparent in large populations, leading to larger estimation errors for *I*_*shuffle*_ in large than small populations. Conversely, the error of the direct estimator of *I*_*shuffle*_ decreases in larger populations (S2 Fig).

We were not able to find an exact analytical expression for an unbiased estimator of *I*_*diag*_ but this scenario is a subcase of the general problem we considered earlier: how to compute information in a dataset B, obtained with an optimal decoder of a dataset A. Here, dataset A is the shuffled data while dataset B corresponds to the original data. Accordingly, we use the same correction as before (Eq (4)), discussed in Materials and Methods, Section 7.

**Fig 5:**
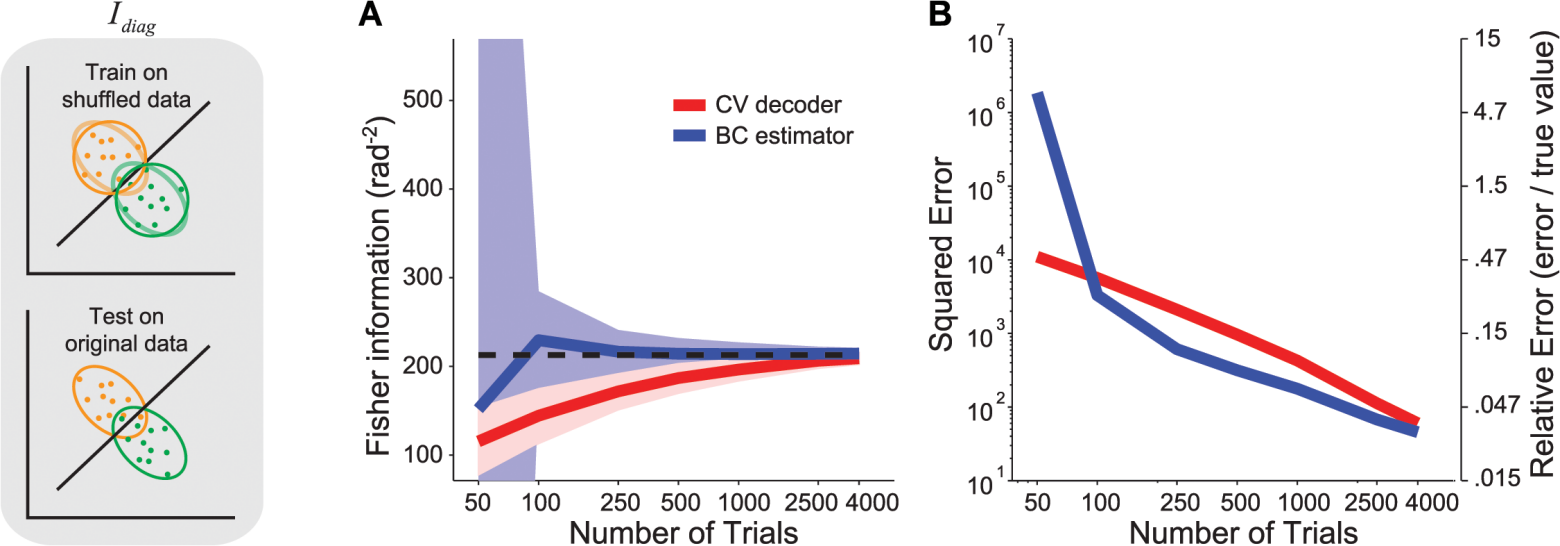
Fisher information estimates when ignoring correlations. Left: Illustration of the scenario. Decoding weights are optimized for the shuffled data, then tested on the original data. The faint lines on the top plot represent the covariance ellipses of the original data, before shuffling. Note that the decision boundary derived from shuffled data is not necessarily optimal for the original data. (**a**) Estimate of the Fisher information obtained by decoding (red) or direct estimation with bias correction (blue). The continuous lines represent the mean, the shaded area represents ±1 std across experiments, computed by bootstrap. (**b**) MSE of the decoder-based estimate (red) and the direct estimator (blue).

In Fig 5, we show the comparison for *I*_*diag*_ and find that the direct estimator is more reliable than the cross-validated decoder for *T* ≥ 2 *N*. For this set of simulations, the cross-over between direct estimator and decoder occurs at a smaller *T* than in the simulations for *I*_*AB*_ (Fig 3B), although this needs not be true in general. Note that the accuracy of the estimator for *I*_*diag*_ (relative error of 6% compared to ground truth, for *T* = 1000) is comparable to those for *I* (namely the information in the original data; relative error of 6% for *T* = 1000) and *I*_*shuffle*_ (relative error of 5% for *T* = 1000), despite the lack of an analytical expression for *I*_*diag*_. In these simulations, the actual values of *I* and *I*_*diag*_ were similar (compare the dashed line in Figs 1 and 5). We therefore repeated the analysis using a different model of noise correlations [12](S1 Text), one which produces a larger gap between *I* and *I*_*diag*_, and found similar results (S3 Fig).

### Evaluations on neural data

So far we have tested the bias-corrected estimators on simulated data, which was generated using a rectified multivariate Gaussian distribution followed by a Poisson step. Even though the analytical form of the bias correction was derived assuming Gaussian variability, our results show that the bias-correction works well for the Gaussian-Poisson model.

To provide a stronger test of the estimation methods, we applied them to data recorded from populations of neurons in the primary visual cortex of an anesthetized macaque monkey. In this experiment, spike count responses were recorded from 52 units to gratings of two different orientations, each presented for 900 trials (Materials and Methods Section 9).

**Fig 6:**
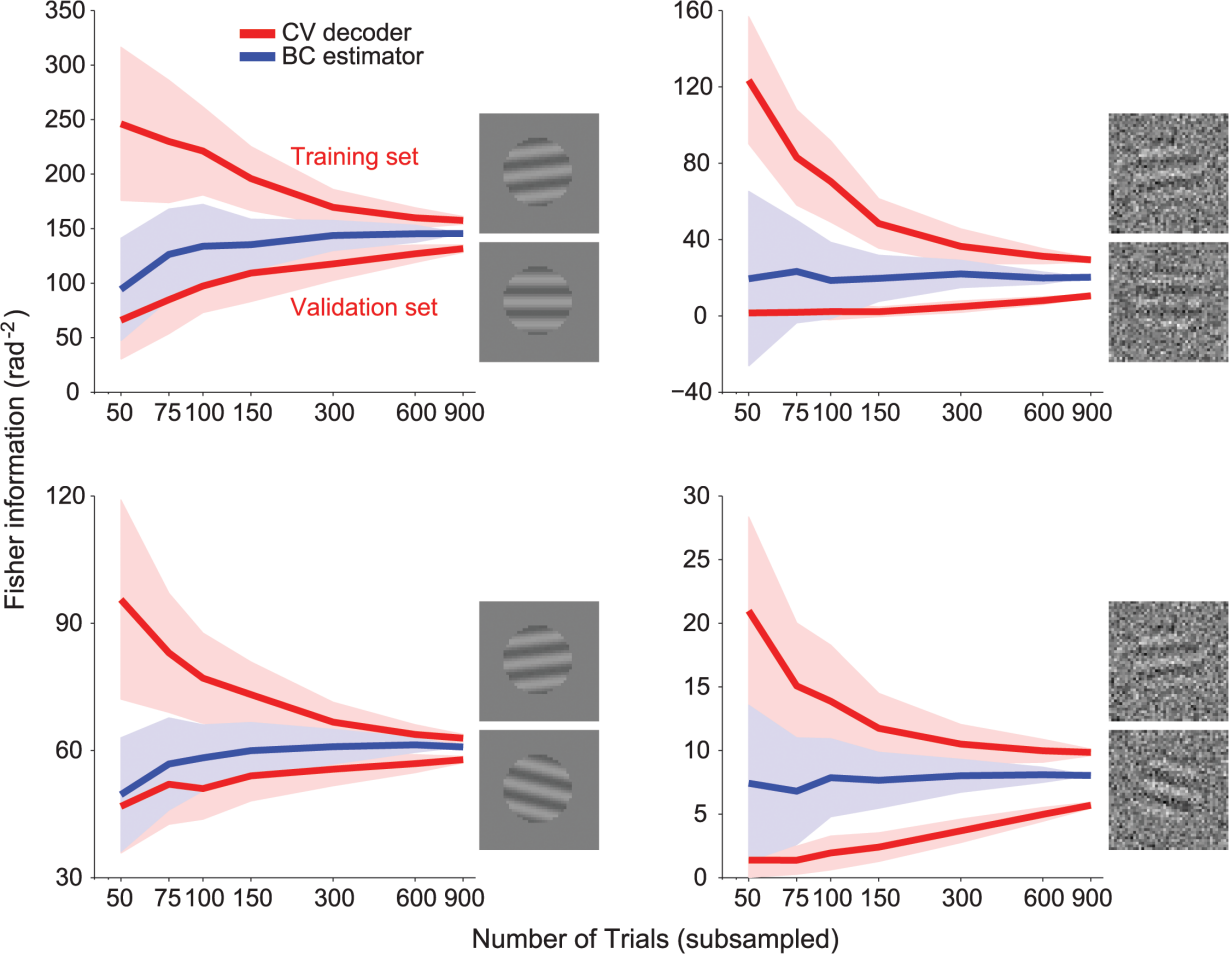
Fisher information estimated from cortical data. Fisher information in a population of *N* = 52 macaque V1 neurons, estimated by decoding (red) or direct estimation (blue). Trials were subsampled 100 times without replacement, except for the last point on the abscissa which included all (*T* = 900) trials. The continuous lines represent the mean, the shaded area represents ±1 std across samples, computed by bootstrap. Stimulus orientations were spaced by 7 deg (top row) and 21 deg (bottom row); in the right column, images were masked by white noise on the pixels (for stimulus details, see Materials and Methods Section 9). Population-average firing rates were R = 0.7 spikes/trial (top-left); R = 0.7 spikes/trial (bottom-left); R = 2.2 spikes/trial (top-right); and R = 2.1 spikes/trial (bottom-right). Note that for large orientation differences, the stimuli can be more easily discriminated: Using the bias-corrected estimate at *T* = 900 and the known conversion between Fisher information and percent correct, percent correct with 7 deg separation is 77% (top-left) and 61% (top-right), whereas the corresponding values with 21 deg separation are 92% (bottom-left) and 70% (bottom-right).

We first used a cross-validated decoder to estimate information. Fig 6 (top-left) shows that the decoder’s upper and lower information bounds (obtained from the training and validating sets) diverge for small numbers of trials as was found previously for simulated data (Fig 1B). While the gap between the two bounds is much reduced when using all 900 trials, it remains sizeable (18% of the asymptotic value). In contrast, the information obtained from a bias-corrected estimator is within 10% of its asymptotic value with a little as 100 trials, Moreover, the asymptotic value lies in between the two bounds from the cross-validated decoder indicating that the bias corrected estimate must be close to the true information value. Using the mean between the two bounds for the decoder as ground truth, we also verified that in most conditions the bias-corrected estimator leads to a lower mean squared error (S4 Fig). This outcome is not guaranteed: in principle, were the bias correction not properly calculated, the direct estimator could reach an asymptotic value that differs largely from the asymptotic value of the cross-validated decoder. Thus, this outcome indicates that the bias correction derived under the assumption of Gaussian variability is also accurate for non-Gaussian cortical variability.

We then asked whether the performance improvement for direct estimation over decoding is robust. We expanded the stimulus set, by manipulating two parameters that are known to affect the estimator accuracy (explained in Materials and Methods Section 4, Eq (19)). First, we varied the total amount of information in the population, by manipulating the noise added to the image pixels (different columns in Fig 6). Second, we varied the difference between the two stimulus values, using *d θ* = {7, 21} degrees (different rows in Fig 6). Fig 6 shows that the gap between decoder estimates on training and validation sets remained sizeable (between 9% and 95% of the asymptotic value) at 900 trials in all conditions. In contrast, the direct estimator reached within 10% of its asymptotic value, with 50 to 150 trials. We compared also the direct and decoding-based estimators of *I*_*shuffle*_ and *I*_*diag*_, and found for the cortical data (S5 Fig) results analogous to the simulations of Figs 4 and 5, thus confirming robustness to realistic deviations from the assumptions of the bias-corrected estimators.

### Robustness to deviations from Gaussianity at low spike counts

The performance of the direct estimator for data simulated using the Gaussian-Poisson model and for non-Gaussian cortical data indicates that the direct estimator is robust to realistic deviations from the Gaussianity assumption. The response variability is expected to deviate further from Gaussian at low spike counts per trial, which can be manipulated both in the simulated data as well as in the cortical data by reducing the observation window.

Note that there is an important difference between linear and full Fisher information: For low spike counts or short time windows there is no guarantee that there exists an efficient (non-linear) estimator reaching the Cramer-Rao bound [23–25]. In contrast, linear Fisher information for discrimination is *defined* to be the inverse minimum stimulus-conditioned variance of a linear estimator which is unbiased for the two presented stimuli. This linear estimator can always be constructed given the tuning curves and noise covariance matrix and by definition there is never a discrepancy between optimal linear estimator variance and inverse linear Fisher information as in the non-linear case. Also, linear Fisher information is defined for general response distributions with existing first and second moments and does not require the assumption of Gaussian response variability.

**Fig 7:**
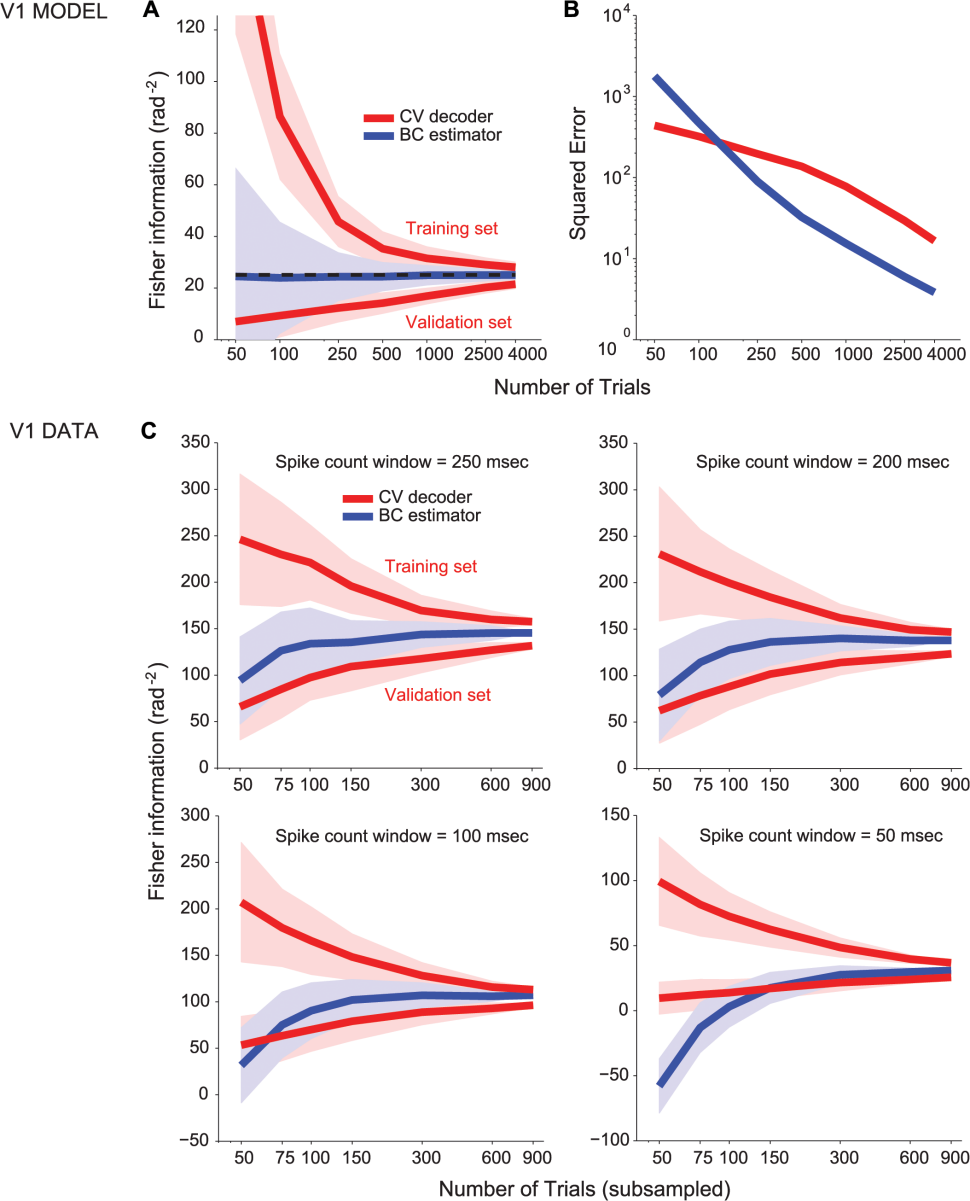
Comparison of estimators at low spike counts. (**a,b**) Simulated data with model parameters identical to Table 1 and Table 2 except for *g* = 1 (corresponding to 0.8 spikes per neuron per trial on average). (**a**) Estimate of the Fisher information obtained by decoding (red) or direct estimation with bias correction (blue). The continuous lines represent the mean, the shaded area represents ±1 std across experiments, computed by bootstrap. (**b**) MSE of the decoder-based estimate (red) and the direct estimator (blue). (**c**) Evaluation on the neural data of Fig 6, in the condition of stimulus orientation spaced by 7 deg, without pixel noise in the image. Each panel corresponds to a different spike count window, reported at the top of the panel, starting 30 ms after stimulus onset. The top-left panel (250ms window) is identical to the top-left panel of Fig 6. The color code is identical to Fig 6.

In Fig 7A and 7B, we compare the performance of the direct estimator with the cross-validated decoding methods at low spike counts for simulated data. The parameters of the plot are the same as in Fig 2A, except for the average tuning amplitude, which we set to *g* = 1 instead of *g* = 30, corresponding to 0.8 spikes per neuron per trial (compared to 11.3 spikes per neuron per trial in Fig 2A). At low spike counts, we cannot compute analytically the ground truth information accurately in our model, due to the approximation used to account for the rectification. We have used instead the estimate obtained with a large number of trials (*T* = 100,000). We find that as soon as *T* ≥ 3*N*, the direct estimator is more reliable than the decoding estimate. Similar results are obtained for a model of Von Mises tuning curves and independent Poisson variability, in which the ground truth can be calculated (S6 Fig).

In Fig 7C we test the robustness for cortical data at low spike counts by successively shortening the observation window. For the original observation widow (250ms, top-left panel of Fig 6) the average count is 0.7 spikes per neuron per trial, which is reduced to 0.07 spikes per neuron per trial for the smallest observation window of 50ms in Fig 7C. For such a small observation windows we observe that the direct estimator does get biased at small numbers of trials. This is due to the large deviation from Gaussianity: In particular, the abundance of trials where not a single spike was fired by any neuron (19% of the trials for a 50ms window, compared to 0.07% of trials for 250ms window) implies that more trials are required to properly estimate the covariance matrix. For extremely low spike counts and scarce data, cross-validated decoding might lead to better results than the direct estimator. However, the direct estimator remains asymptotically unbiased regardless of window size, and even for a 100ms window (average spike count of 0.24, with 4% silent trials) it is unbiased with as little as *T* ≥ 2*N*.

## Discussion

We have presented a fast and accurate method to estimate the amount of information about an encoded stimulus in a correlated neural population. As recently pointed out [9], estimating the tuning curve derivatives and covariance matrix from the data, and then applying the equation that defines Fisher information, Eq (1), leads to large biases. We have shown that the bias of the direct estimator can be predicted exactly, and we have demonstrated that correcting for this bias leads to accurate estimates of the true information in the population. Using both realistic simulations as well as experimental data, we have shown that our bias-corrected estimator largely outperforms the current state of the art methods, based on decoding. Furthermore, while training a decoder requires a typically lengthy numerical minimization, the method we proposed only requires a matrix inversion, and is therefore orders of magnitude faster. We have also derived an analytical expression for the variance of the estimator, and extended our bias-correction method to the widely studied cases of independent populations obtained by shuffling the data, and of a factorized decoder (i.e. decoding correlated data under the assumption that there are no correlations). As datasets of increasing size become available, our method will provide an invaluable tool to explore quantitatively the relation between the neural code and behavioral variability.

Our bias correction method assumes Gaussian variability of neuronal responses, an assumption that is often violated by experimental data. Therefore, we compared our estimator and decoding on neural data recorded from macaque primary visual cortex. In this case we do not have access to the true information; however, by subsampling trials, we showed that the two methods give consistent results in the limit of large numbers of trials, but our estimator is systematically more accurate, except for very low spike counts when only a few trials are available.

Why is our direct estimator more accurate than decoding? Both training a decoder, and assessing its performance, require large amount of data. Therefore, to use decoding, the available trials need to be split in similar proportions between the training set and validation set; in addition, early stopping requires further splitting of the training set, in order to monitor and prevent overfitting. As a consequence, decoding-based methods have reduced statistical power, compared to direct estimation. One way to mitigate this issue is to use model-based regularization (e.g. variational Bayes logistic regression [26] or L_2_ regularization), to avoid splitting the training set, and leave-one-out cross validation to maximize the size of the training set. However, these approaches also rely on assumptions about the data that are not always met, and are subject to overfitting for finite datasets. We found that the performance of these alternative decoding methods was not systematically better than early stopping (S7 Fig). All three decoding methods performed significantly worse than the bias-corrected estimator and had run times between 1 and 4 orders of magnitude larger than the bias-corrected estimator (S7 Fig).

Our derivation of an analytical expression for the variance of the direct, bias-corrected estimator allows one to draw exact error bars without relying on bootstrapping methods. It also allowed us to understand limiting conditions under which the estimation error explodes. First we found that the variance of the bias-corrected estimator diverges for *T* = (*N* + 5) / 2. This is the lower bound on the number of trials which need to be present for the estimator to be useable. Currently, if fewer trials than this lower bound are available, decoding remains the only available method, although the estimates it provides may be highly inaccurate. Extending our direct estimator to this case is an important direction for future work.

Second, we found that the estimation error becomes large if the difference of the presented stimuli, *d θ*, is small relative to the inverse square root of the true Fisher information $1/\sqrt{I}$ (see Eq (19)). The reason is that the smaller *d θ* is, the noisier the estimation of the tuning curve derivative **f**′ will be. Conversely, if the aim is to measure the information available in a fine discrimination task, *d θ* cannot be too large either: This will lead to a bias in the estimation for non-linear tuning curves, because $\frac{\Delta f}{d\theta }\neq f\prime$. If the aim is to estimate information in a fine discrimination tasks, the experimental choice of *d θ* must strike a balance between these two constraints. Note however that, for any fixed *d θ* our estimator will still have lower variance and less underestimation than a linear decoder, when *T* > *N*. Hence, the choice of *d θ* should not influence the decision whether to use the decoder or the direct estimator. The direct estimator is also a better option for coarse discrimination tasks, as long as one is interested in linear Fisher information. The only difference in the case of coarse discrimination compared to fine discrimination is that the derivative of the tuning curves should be replaced with $\frac{\Delta f}{d\theta }$.

For investigators interested in estimating Fisher information, as opposed to linear Fisher information, other techniques must be used such as cross-validated nonlinear decoders. However, training a non-linear decoder from limited data will be even more difficult than training a linear decoder and will most likely lead to biased estimates of Fisher information. We do not know yet whether it is possible to obtain direct unbiased estimates of Fisher information, as we have described here for linear Fisher information.

Our results apply to the case of linear Fisher information about a continuous stimulus in a fine or coarse discrimination task. However, when studying the neural code in higher cortical areas, typically higher-level tasks are used such as object recognition, which involve a classification between multiple discrete classes [27]. Extending our approach to such multiclass classification is another important direction for future work.

## Materials and Methods

### Ethics statement

All procedures were approved by the Albert Einstein College of Medicine at Yeshiva University and followed the guidelines in the United States Public Health Service Guide for the Care and Use of Laboratory Animals.

#### 1. Derivation of linear Fisher information

Linear Fisher information is defined to be the inverse variance of the locally optimal unbiased linear decoder [4]. Given two presented stimuli *θ* ^+^ = *θ* + *dθ* and *θ* ^−^ = *θ*−*dθ* in a fine discrimination task one constructs a locally linear estimator by the relation

$${\hat{\theta }}_{w}(r)=\theta +w^{T}\cdot (r-\frac{f(\theta^{+})+f(\theta^{-})}{2})$$

We would like to minimize the variance of this estimator while ensuring it to be unbiased for the two presented stimuli. This yields

$$w_{opt}=\frac{\Sigma^{-1}f\prime }{f\prime^{T}\Sigma^{-1}f\prime }$$ (7)

and

$$\text{Var}({\hat{\theta }}_{w_{opt}}(r)|\theta^{i})=\frac{1}{f\prime^{T}\Sigma^{-1}f\prime }$$

In analogy with the Cramer-Rao bound, linear Fisher information is defined to be the inverse of this variance:

$$I\equiv \frac{1}{\text{Var}({\hat{\theta }}_{w_{opt}}(r)|\theta^{i})}=f\prime^{T}\Sigma^{-1}f\prime$$

Given the tuning curves and noise covariance matrix one can always find the optimal discrimination weights given by Eq (7). As a consequence, linear Fisher information is always attainable, unlike the full, non-linear Fisher information. Note also that this derivation does not include any assumptions about Gaussian response variability.

Furthermore, the above derivation of linear Fisher information can be straightforwardly extended to coarse discrimination and to the case of different noise covariance matrices at the two presented stimuli. In this case linear Fisher information is given by

$$I=\frac{df\;{\bar{\Sigma }}^{-1}\;df}{{\left(\theta^{+}-\theta^{-}\right)}^{2}}\;,$$

where *d* **f** = **f**(*θ* ^+^)−**f**(*θ* ^−^) and $\bar{\Sigma }=\frac{1}{2}\left(\Sigma (\theta^{+})+\Sigma (\theta^{-})\right)$. For ease of presentation we focus on fine discrimination and equal covariances in the remainder of the Material & Methods.

#### 2. Estimating Fisher information by decoding

The method used most frequently to estimate Fisher information in neural population data is based on decoding. Given population responses to *T* trials of stimulus *θ* ^+^ = *θ* + *dθ* and *T* trials of stimulus *θ* ^−^ = *θ*−*dθ*, one uses a locally optimal unbiased linear decoder to estimate the stimulus value, and takes the inverse of the variance of the estimate of the stimulus to be the Fisher information [4].

Finding the optimal decoder can be formalized as a regression problem: Find the decoding weights vector **w** that minimizes the following squared error

$$E=\sum_{t=1}^{2T}{\left[\theta_{t}-\langle \theta \rangle -w^{T}\cdot \left(r_{t}-\langle r\rangle \right)\right]}^{2}$$

where *θ*_*t*_ and **r**_*t*_ denote, respectively, the true stimulus value and the population response on trial *t*, and 〈·〉 denotes the average across all trials. Since all estimates are based on finite number of trials, this method leads to overfitting, which in turn leads to overestimation of the true Fisher information [9] (see Fig 1A). Therefore, cross-validation must be used to assess decoder performance and some form of regularization is required to mitigate overfitting. Here, following Moreno et al. [9], we use early stopping (we also considered alternative approaches to regularization and found similar results, S7 Fig). We split the data in three sets (training, test, and validation) of approximately equal size, and update the decoding weights by gradient descent on the training set

$$\frac{\partial w}{\partial \tau }\propto -\frac{\partial E_{TR}}{\partial w}$$

where *E*_*TR*_ is the squared error on the training set. We initialize the weights randomly, and update them until the test set error, denoted *E*_*TE*_, starts to increase. This is when its derivative ∂*E*_*TE*_ / ∂*τ* changes sign from negative to positive, where

$$\frac{\partial E_{TE}}{\partial \tau }={\left(\frac{\partial E_{TE}}{\partial w}\right)}^{T}\cdot \frac{\partial w}{\partial \tau }\propto -{\left(\frac{\partial E_{TE}}{\partial w}\right)}^{T}\cdot \frac{\partial E_{TR}}{\partial w}$$

Once the optimization terminates, the Fisher information is estimated on the validation set

$$I_{\text{Early Stopping}}={\left[\frac{w^{T}\cdot \left(\langle r_{VAL}\left(\theta^{+}\right)\rangle -\langle r_{VAL}\left(\theta^{-}\right)\rangle \right)}{\theta^{+}-\theta^{-}}\right]}^{2}\frac{2}{w^{T}\cdot S_{VAL}\left(\theta^{+}\right)\cdot w+w^{T}\cdot S_{VAL}\left(\theta^{-}\right)\cdot w}$$ (8)

where 〈**r**_*VAL*_(*θ* ^±^)〉 denotes the average population responses, and *S*_*VAL*_(*θ* ^±^) the sample covariance matrices, computed from all trials in the validation set corresponding to either stimulus *θ* ^+^ or *θ* ^−^. Note that the first term on the r.h.s. corrects for biases of the decoder, e.g. due to a wrong scaling of **w**.

#### 3. Bias-corrected estimator of full Fisher information

Here we show that the estimator in Eq (2) for Fisher information is unbiased assuming Gaussian variability. In this case the response distribution in trials *t* = 1…*T* to stimuli *θ* ± *d θ* is given by the multivariate Gaussian

$$r_{t}^{\pm }∼N\left(f\left(\theta \pm d\theta \right),\Sigma \right)\approx N\left(f(\theta )\pm d\theta f\prime \left(\theta \right),\Sigma \right),$$ (9)

where we assume the difference between the two presented stimuli *d θ* to be small enough that we can linearly expand the tuning curve and neglect the change in covariance as a function of *θ*. The empirical mean and covariance for each presented stimulus is given by

$$\begin{array}{l} \mu^{\pm }=\frac{1}{T}\sum_{t=1}^{T}r_{t}^{\pm }\\ S^{\pm }=\frac{1}{T-1}\sum_{t=1}^{T}\left(r_{t}^{\pm }-\mu^{\pm }\right){\left(r_{t}^{\pm }-\mu^{\pm }\right)}^{T} \end{array}$$

This allows us to construct unbiased estimators of both **f**′(*θ*) and Σ:

$$\begin{array}{c} \frac{d\mu }{d\theta }\equiv \frac{\mu^{+}-\mu^{-}}{d\theta };\;\langle \frac{d\mu }{d\theta }\rangle =f\prime (\theta )\\ S\equiv \frac{1}{2}\left(S^{+}+S^{-}\right);\;\langle S\rangle =\Sigma \end{array}$$ (10)

Since the linear Fisher information is a non-linear function of **f**′(*θ*) and Σ the naive estimator

$${\hat{I}}_{nv}=\frac{d\mu^{T}}{d\theta }S^{-1}\frac{d\mu }{d\theta }$$ (11)

will have a bias which we will now calculate. For that we make use of the fact that the sampling distributions of the empirical mean and covariance given *T* trials are given by

$$\begin{array}{l} \mu^{\pm }∼N\left(f\left(\theta \right)\pm d\theta f\prime \left(\theta \right),\frac{\Sigma }{T}\right)\\ S^{\pm }∼W_{N}\left(\frac{\Sigma }{T-1},T-1\right) \end{array}$$ (12)

where *W*_*p*_(*V*, *n*) is the *p*-dimensional Wishart distribution with *n* degrees of freedom [14]. Consequently, the unbiased estimators in Eq (10) are sampled from

$$\begin{array}{l} \frac{d\mu }{d\theta }∼N\left(f\prime (\theta ),\frac{2\Sigma }{Td\theta^{2}}\right)\\ \;S∼W_{N}\left(\frac{\Sigma }{2(T-1)},2(T-1)\right) \end{array}$$ (13)

If Σ is invertible and *N* < 2(*T*−1), *S* will be invertible with probability 1. The expectation value of its inverse is given by [14]

$$\langle S^{-1}\rangle =\Sigma^{-1}\frac{2T-2}{2T-N-3}$$

The second result we need is that the sampling distributions of mean and covariance of a Gaussian are independent (see e.g. [28]). It follows that the expectation value of the naive estimator Eq (11) is given by

$$\begin{array}{l} \langle {\hat{I}}_{nv}\rangle =\langle \frac{d\mu^{T}}{d\theta }S^{-1}\frac{d\mu }{d\theta }\rangle =Tr\left(\langle \frac{d\mu }{d\theta }\frac{d\mu^{T}}{d\theta }S^{-1}\rangle \right)=Tr\left(\langle \frac{d\mu }{d\theta }\frac{d\mu^{T}}{d\theta }\rangle \langle S^{-1}\rangle \right)\\ \;=\frac{2T-2}{2T-N-3}Tr\left((f\prime f\prime^{T}+\frac{2\Sigma }{Td\theta^{2}})\Sigma^{-1}\right)=\frac{2T-2}{2T-N-3}\left(I+\frac{2N}{Td\theta^{2}}\right) \end{array}$$

Correcting for this bias yields the expression for the bias-corrected estimator Eq (2). Note that the sampling distributions of mean and covariance are independent if and only if the underlying distribution is Gaussian. Measuring the independence of sample mean and covariance in real data with a relevant number of trials is very difficult since it would require repeating the same experiment many times. It is easier to determine whether the response distribution is close to Gaussian, which is the case only at high spike count. The fact that the bias-corrected estimator is robust to deviations from Gaussianity at low spike count implies that it is also robust to the dependence of the sampling distribution of mean and covariance.

Note that the correction in Eq (2) can lead occasionally to negative estimates of information. This will only happen when the estimate is very noisy, in which case the error bars are expected to be of similar magnitude as the actual estimate. As we show next, we can also derive an analytical prediction for the variance of the estimator, which allows us to draw error bars even for a single experiment.

#### 4. Variance of the bias-corrected estimator

Due to finite-sampling variability, the estimator in Eq (2) evaluated in a single experiment with *T* trials can be interpreted as a random draw from its sampling distribution. In the following we compute analytically the variance of this sampling distribution which allows calculating error bars on the measurements. The estimator in Eq (2) can be rewritten as

$${\hat{I}}_{bc}=\sum_{i,j}X_{ij}{\hat{S}}_{ij}^{-1}-\frac{2N}{Td\theta^{2}}$$

where we have defined

$$\begin{array}{l} X_{ij}=\frac{d\mu_{i}}{d\theta }\frac{d\mu_{j}}{d\theta }\\ {\hat{S}}^{-1}=\frac{2T-N-3}{2T-2}S^{-1} \end{array}$$ (14)

Since the sampling distributions of *X*_*ij*_ and ${\hat{S}}_{ij}^{-1}$ are independent the variance of ${\hat{I}}_{bc}$ is given by

$$Var\;{\hat{I}}_{bc}=\sum_{i,j,k,l}\langle X_{ij}\rangle \langle X_{kl}\rangle Cov({\hat{S}}_{ij}^{-1},{\hat{S}}_{kl}^{-1})+Cov(X_{ij},X_{kl})\left(\langle {\hat{S}}_{ij}^{-1}\rangle \langle {\hat{S}}_{kl}^{-1}\rangle +Cov({\hat{S}}_{ij}^{-1},{\hat{S}}_{kl}^{-1})\right)$$ (15)

The expectation values are given by

$$\begin{array}{l} \langle X_{ij}\rangle =f_{i}\prime f_{j}\prime +\gamma \Sigma_{ij}\\ \langle {\hat{S}}_{ij}^{-1}\rangle =\Sigma_{ij}^{-1} \end{array}$$ (16)

where we have defined *γ* = 2 / (*Tdθ* ^2^). The covariance of *X*_*ij*_ can be calculated using the higher moments of the Gaussian distribution in Eq (13),

$$\begin{array}{l} Cov\left(X_{ij},X_{kl}\right)=\gamma \left(f_{i}\prime f_{k}\prime \Sigma_{jl}+f_{j}\prime f_{l}\prime \Sigma_{ik}+f_{i}\prime f_{l}\prime \Sigma_{jk}+f_{j}\prime f_{k}\prime \Sigma_{il}\right)\\ \;+\gamma^{2}\left(\Sigma_{ik}\Sigma_{jl}+\Sigma_{il}\Sigma_{jk}\right) \end{array}$$ (17)

The covariance of ${\hat{S}}_{ij}^{-1}$ can be found using well-known expressions for the inverse Wishart distribution [14]

$$Cov\left({\hat{S}}_{ij}^{-1},{\hat{S}}_{kl}^{-1}\right)=\alpha \Sigma_{ij}^{-1}\Sigma_{kl}^{-1}+\beta \;\left(\Sigma_{ik}^{-1}\Sigma_{jl}^{-1}+\Sigma_{il}^{-1}\Sigma_{jk}^{-1}\right)$$ (18)

where

$$\alpha =\frac{2}{\left(2T-N-2\right)\left(2T-N-5\right)}\;,\;\beta =\frac{2T-N-3}{\left(2T-N-2\right)\left(2T-N-5\right)}$$

Combining Eq (16), Eq (17) and Eq (18) we can calculate Eq (15) in a tedious, but straightforward calculation. The result is

$$Var\;{\hat{I}}_{bc}=\frac{2I^{2}}{2T-N-5}\left(1+\frac{4(2T-3)}{TId\theta^{2}}+\frac{4N(2T-3)}{T^{2}I^{2}d\theta^{4}}\right)$$ (19)

This formula provides several insights. First, one can see that the variance of the bias-corrected estimator diverges for *T* = (*N* + 5) / 2. This is the lower bound on the number of trials which need to be present for the estimator to be useable. Second, one can see that the second and third term in Eq (19) are large if the difference of the presented stimuli, *d θ*, is small relative to the inverse square root of the true Fisher information $1/\sqrt{I}$. The reason is that for small *d θ* the estimate of the tuning curve derivative **f**′ will be noisy. Conversely, if *d θ* is too large, one will introduce a bias in the estimation if the true tuning curve is non-linear. The right choice of *d θ* involves finding a tradeoff between these two constraints.

#### 5. Bias-corrected estimator of *I*_*shuffle*_

An unbiased estimator for *I*_*shuffle*_ can be derived in a similar way. Here we assume that the covariance is diagonal, $\Sigma_{ij}=\sigma_{i}^{2}\delta_{ij}$. The unbiased estimator for the variances on the diagonal is given by

$$\begin{array}{c} s_{i}^{2}=\frac{1}{2}\left({\left(s_{i}^{+}\right)}^{2}+{\left(s_{i}^{-}\right)}^{2}\right)\\ {\left(s_{i}^{\pm }\right)}^{2}=\frac{1}{T-1}\sum_{t=1}^{T}{\left(r_{i,t}^{\pm }-\mu_{i}^{\pm }\right)}^{2} \end{array}$$

Similarly we can construct the naive estimator

$${\hat{I}}_{nv,shuffle}=\sum_{i}\frac{{\left(d\mu_{i}/d\theta \right)}^{2}}{s_{i}^{2}},$$ (20)

whose bias we will again calculate. The sampling distribution of the diagonal variance is given by a Gamma (or chi-square) distribution:

$$s_{i}^{2}∼\Gamma \left(\alpha =T-1,\beta =\frac{T-1}{\sigma_{i}^{2}}\right)$$

The distribution of the empirical mean is identical to the one in Eq (12). The expectation value of the inverse variance is given by

$$\langle \frac{1}{s_{i}^{2}}\rangle =\frac{T-1}{(T-2)\sigma_{i}^{2}}$$

Using again the independence of sample mean and variance, the expectation value of the naive estimator Eq (20) is given by

$$\langle {\hat{I}}_{nv,shuffle}\rangle =\sum_{i}\langle {\left(\frac{d\mu_{i}}{d\theta }\right)}^{2}\rangle \langle \frac{1}{s_{i}^{2}}\rangle =\sum_{i}\left(f_{i}\prime^{2}+\frac{2\sigma_{i}^{2}}{Td\theta^{2}}\right)\frac{T-1}{(T-2)\sigma_{i}^{2}}=\frac{T-1}{T-2}\left(I_{shuffle}+\frac{2N}{Td\theta^{2}}\right)$$

Correcting for the bias yields the estimator Eq (6) for *I*_*shuffle*_.

#### 6. Bias-corrected estimator for a suboptimal decoder

Here we set out to find an unbiased estimator for the general case of a decoder optimized to dataset A and tested on dataset B. We assume that A and B are generated by neural populations with independent covariance matrices and tuning curves. The optimal decoding weights for dataset A are

$$w_{A}\propto \Sigma_{A}^{-1}\cdot f{'}_{A},$$ (21)

and the information that can be extracted by such decoder from dataset B is

$$I_{AB}=\frac{{\left(f{'}_{B}^{T}\cdot w_{A}\right)}^{2}}{w_{A}^{T}\cdot \Sigma_{B}\cdot w_{A}}=\frac{{\left(f{'}_{B}^{T}\cdot \Sigma_{A}^{-1}\cdot f{'}_{A}\right)}^{2}}{f{'}_{A}^{T}\cdot \Sigma_{A}^{-1}\cdot \Sigma_{B}\cdot \Sigma_{A}^{-1}\cdot f{'}_{A}}.$$ (22)

We focus separately on the biases in the naïve estimates of the numerator and denominator, and correct for them. This will not guarantee that the full expression is unbiased since the division by the denominator is a non-linear transformation. Furthermore, all three terms in the numerator are independent (since the tuning curves for populations A and B are assumed independent) hence we correct for their biases individually (more specifically, for the covariance inversion) but we neglect the bias due to the squaring in the numerator. However both the numerator and denominator are one-dimensional quantities, whose variances are of order O(1/T). Therefore we expect that the size of the biases due to squaring and division is of order O(1/T) rather than O(N/T) for the naive estimator.

Focusing now on the denominator, a first step towards removing the bias is to use the bias-corrected estimator ${\hat{S}}_{A}^{-1}$ defined in Eq (14) rather than the naive estimator. This will however only remove part of the bias, since ${\hat{S}}_{A}^{-1}$ appears twice in the denominator. The bias is given by

$$\begin{array}{l} \langle \frac{d\mu_{A}^{T}}{d\theta }\cdot {\hat{S}}_{A}^{-1}\cdot S_{B}\cdot {\hat{S}}_{A}^{-1}\cdot \frac{d\mu_{A}}{d\theta }\rangle =Tr\left(\langle \frac{d\mu_{A}}{d\theta }\cdot \frac{d\mu_{A}^{T}}{d\theta }\rangle \langle {\hat{S}}_{A}^{-1}\cdot S_{B}\cdot {\hat{S}}_{A}^{-1}\rangle \right)\\ \;=Tr\left(\frac{2}{Td\theta^{2}}\Sigma_{A}\langle {\hat{S}}_{A}^{-1}\cdot S_{B}\cdot {\hat{S}}_{A}^{-1}\rangle \right)+Tr\left(f{'}_{A}\cdot f{'}_{A}^{T}\langle {\hat{S}}_{A}^{-1}\cdot S_{B}\cdot {\hat{S}}_{A}^{-1}\rangle \right) \end{array}$$ (23)

In order to evaluate $\langle {\hat{S}}_{A}^{-1}\cdot S_{B}\cdot {\hat{S}}_{A}^{-1}\rangle$, we make use of the basic fact that

$$\langle {\left({\hat{S}}_{A}^{-1}\right)}_{ij}{\left({\hat{S}}_{A}^{-1}\right)}_{kl}\rangle ={\left(\Sigma_{A}^{-1}\right)}_{ij}{\left(\Sigma_{A}^{-1}\right)}_{kl}+Cov\left({\left({\hat{S}}_{A}^{-1}\right)}_{ij},{\left({\hat{S}}_{A}^{-1}\right)}_{kl}\right),$$ (24)

where the covariance of ${\hat{S}}_{A}^{-1}$ can be found as in Eq (18). After a lengthy but straightforward calculation, we find that

$$\begin{array}{l} \langle {\hat{S}}_{A}^{-1}\cdot S_{B}\cdot {\hat{S}}_{A}^{-1}\rangle =\\ \left[1+\frac{2T-N-1}{\left(2T-N-2\right)\left(2T-N-5\right)}\right]\Sigma_{A}^{-1}\cdot \Sigma_{B}\cdot \Sigma_{A}^{-1}+\frac{2T-N-3}{\left(2T-N-2\right)\left(2T-N-5\right)}Tr\left(\Sigma_{B}\cdot \Sigma_{A}^{-1}\right)\Sigma_{A}^{-1} \end{array}$$ (25)

It follows that

$$\langle \frac{d\mu_{A}^{T}}{d\theta }\cdot {\hat{S}}_{A}^{-1}\cdot S_{B}\cdot {\hat{S}}_{A}^{-1}\cdot \frac{d\mu_{A}}{d\theta }\rangle =\lambda +\rho +\left[1+\frac{2T-N-1}{\left(2T-N-2\right)\left(2T-N-5\right)}\right]f{'}_{A}^{T}\cdot \Sigma_{A}^{-1}\cdot \Sigma_{B}\cdot \Sigma_{A}^{-1}\cdot f{'}_{A}$$ (26)

Where

$$\begin{array}{l} \lambda =\frac{2}{Td\theta^{2}}\left[1+\frac{2T-N-1+N\left(2T-N-3\right)}{\left(2T-N-2\right)\left(2T-N-5\right)}\right]Tr\left(\Sigma_{A}^{-1}\cdot \Sigma_{B}\right)\\ \rho =\frac{2T-N-3}{\left(2T-N-2\right)\left(2T-N-5\right)}Tr\left(\Sigma_{A}^{-1}\cdot \Sigma_{B}\right)I_{A} \end{array}$$ (27)

and *I*_*A*_ is the true information in population A.

Eq (26) provides an expression for the expected bias. In order to remove such bias from the estimator of *I*_*AB*_, we first need an unbiased estimate the bias itself, which we obtain by substituting, in Eqs (26) and (27), ${\hat{I}}_{bc,A}$ for *I*_*A*_; and *Ŝ* ^−1^ for the inverse covariance Σ^−1^.

As a result, the bias-corrected estimator is given by

$${\hat{I}}_{bc,AB}=\left[\frac{{\left(\frac{d\mu_{B}^{T}}{d\theta }\cdot {\hat{S}}_{A}^{-1}\cdot \frac{d\mu_{A}}{d\theta }\right)}^{2}}{\frac{d\mu_{A}^{T}}{d\theta }\cdot {\hat{S}}_{A}^{-1}\cdot S_{B}\cdot {\hat{S}}_{A}^{-1}\cdot \frac{d\mu_{A}}{d\theta }-\hat{\lambda }-\hat{\rho }}\right]\left(1+\frac{2T-N-1}{\left(2T-N-2\right)\left(2T-N-5\right)}\right),$$ (28)

Where

$$\begin{array}{l} \hat{\lambda }=\frac{2}{Td\theta^{2}}\left[1+\frac{2T-N-1+N\left(2T-N-3\right)}{\left(2T-N-2\right)\left(2T-N-5\right)}\right]Tr\left({\hat{S}}_{A}^{-1}\cdot S_{B}\right)\\ \hat{\rho }=\frac{2T-N-3}{\left(2T-N-2\right)\left(2T-N-5\right)}Tr\left({\hat{S}}_{A}^{-1}\cdot S_{B}\right){\hat{I}}_{bc,A} \end{array}$$

#### 7. Bias-corrected estimator of *I*_*diag*_

The unbiased estimator in Eq (28) was derived under the assumption that datasets A and B are generated by populations with independent covariance matrices and tuning curve derivatives. However, estimating *I*_*diag*_ corresponds to the case that datasets A and B are generated by populations with the same tuning curves derivatives, hence an additional correction is required for the numerator of Eq (22), analogous to the one in Eq (2), leading to

$${\hat{I}}_{bc,AB}=\left[\frac{{\left(\frac{d\mu_{B}^{T}}{d\theta }\cdot {\hat{S}}_{A}^{-1}\cdot \frac{d\mu_{A}}{d\theta }-\frac{2N}{Td\theta^{2}}\right)}^{2}}{\frac{d\mu_{A}^{T}}{d\theta }\cdot {\hat{S}}_{A}^{-1}\cdot S_{B}\cdot {\hat{S}}_{A}^{-1}\cdot \frac{d\mu_{A}}{d\theta }-\hat{\lambda }-\hat{\rho }}\right]\left(1+\frac{2T-N-1}{\left(2T-N-2\right)\left(2T-N-5\right)}\right)$$ (29)

Note that in this case, *S*_*B*_ represents the sample covariance of the original data, whereas ${\hat{S}}_{A}^{-1}$ represents the bias-corrected inverse covariance of the shuffled data (i.e. it includes residual correlations due to shuffling a finite number of trials). Note also that, since *S*_*A*_ and *S*_*B*_ are derived from the same data, before and after shuffling, they are not exactly independent. Hence, ${\langle {\hat{S}}_{A}^{-1}\cdot S_{B}\cdot {\hat{S}}_{A}^{-1}\rangle }_{il}\approx \sum_{j,k}\langle {\hat{S}}_{A,ij}^{-1}{\hat{S}}_{A,ikl}^{-1}\rangle \langle S_{B,jk}\rangle$ holds only approximately (i.e. neglecting 3^rd^ order terms). In practice, as shown in the Results, this approximation provides good results.

#### 8. Models of correlated neural populations

The simulations are based on a bank of orientation-selective filters. The inputs to the network are 32×32 pixel Gabor patches corrupted by additive white noise with variance $\sigma_{0}^{2}$. The Gabor patches are defined by:

$$\text{J}{\left(\theta \right)}_{\left(x,y\right)}=c\;\text{exp}\left(-\frac{x^{2}+y^{2}}{2\sigma^{2}}\right)\text{cos}\left(\frac{2\pi }{\lambda }\left(x\;\text{cos}\;\theta +y\;\text{sin}\;\theta \right)+\phi \right)$$ (30)

where (*x*, *y*) are the coordinates of the image, *c* the carrier contrast, *σ* is the size of the Gaussian envelope, *λ* the preferred spatial wavelength, *θ* the preferred orientation, and *ϕ* the phase offset of the Gabor filter (parameters values are specified in Table 1). The inputs to the network, after the addition of noise, are:

$$\tilde{J}∼N(J(\theta ),\sigma_{0}^{2}1_{P})$$ (31)

where *P* is the image length in pixels and **1**_*P*_ is the identity matrix of size *P* × *P*.

**Table 1:** Parameters of the input images.

Symbol	Meaning	Value
*P*	Side length (pixels)	32
*θ*	Orientation (degrees)	{−7, 0}
*σ*	Gaussian envelope std (degrees)	*P*/5
*λ*	Preferred spatial wavelength (pixel/cycle)	*P*/1.5
*ϕ*	Preferred spatial phase	0
*c*	Michelson contrast	0.75
*σ*_0_	Input noise std	0.2

Model neurons are represented by linear filters whose outputs are half-rectified, and further corrupted by independent Poisson noise. This is a doubly stochastic model, with part of the variability induced by input fluctuations, and part due to the Poisson step. The linear filters are also Gabor patches rescaled to have zero mean and norm 1, and with the same size, wavelength and phase as the image patches (parameter values are provided in Table 2). We denote by **F**_*k*_ the filter representing the *k*-th neuron. Fisher information in a population of such neurons is determined by their tuning curves and covariance matrix, which, ignoring half-rectification, are given by:

$$f_{k}(\theta )=a_{k}F_{k}\cdot J(\theta )$$ (32)

$$\Sigma_{kl}(\theta )=\sigma_{0}^{2}a_{k}a_{l}F_{k}\cdot F_{l}+\delta_{kl}f_{k}(\theta )$$ (33)

with random amplitudes *a*_*k*_ drawn from a log-normal distribution. We fixed the phase offset of the images and neurons to 0, such that the output of the filters is never negative.

**Table 2:** Parameters of the model filters.

Symbol	Meaning	Value
*N*	Number of neurons	50
*P*	RF side length (pixels)	32
*θ*	Preferred orientation (degrees)	$\left[-180:\frac{360}{N}:\frac{180(N-1)}{N}\right]$
*σ*	Gaussian envelope std (pixels)	*P*/5
*λ*	Preferred spatial wavelength (pixel/cycle)	*P*/1.5
*ϕ*	Preferred spatial phase	0
*g*_*k*_	Tuning amplitude	*g*_*k*_ = *ga*_*k*_; *g* = 30; *a*_*k*_ ∼ *LogNormal* (0.25)

We considered a fine discrimination between stimuli *θ*_+_ and *θ*_−_, with *d θ* = 7 degrees. To compute the ground-truth information, we first evaluated the local tuning curve derivatives and covariance:

$$\begin{array}{c} f'=\frac{f(\theta_{+})-f(\theta_{-})}{d\theta }\\ \Sigma =\frac{\Sigma (\theta_{+})+\Sigma (\theta_{-})}{2} \end{array}$$

and then used Eq (1).

To evaluate information with the bias-corrected estimator and the decoder, we generated synthetic population responses from the network. Each simulated experiment comprised T trials per stimulus condition. The number of trials varied between 50 and 4000 (see figures), and for each T we ran 200 experiments. An experiment started by sampling images from Eq (31), then taking the dot product between the images and the neural filters, Eq (32). The filters’ outputs were then half-rectified, and used to define the mean of the Poisson process from which we sampled the spike counts, to produce realistic response variability. Therefore, the spike count of neuron *k* during trial *t* was:

$$r_{k,t}∼Poisson\left({\left\lfloor a_{k}F_{k}\cdot {\tilde{J}}_{t}\right\rfloor }_{+}\right)$$ (34)

Note that in this model, the information in the cortical population cannot exceed the information in the input image, namely $I_{input}=\frac{{\left|J\prime \right|}^{2}}{\sigma_{0}^{2}}$. Therefore, correlations in this model limit information (i.e. the responses contain differential correlations [9]).

In Fig 3, we considered the responses of two populations with different parameters, called population A and B. For population A we used the parameter values of Table 2. For population B, we used filters with smaller Gaussian envelope (*σ* = *P* / 8) and shorter preferred spatial wavelength (*λ* = P / 3).

Note that due to the half-rectification, Eq (32) and Eq (33) are only approximations to the true tuning and covariance. In another set of simulations (described in S1 Text) we considered a different model where the true tuning curves and covariance (and therefore the true information) are known exactly, and verified that the results were unchanged. Specifically, the direct estimator bias and variance were exactly predicted by Eq (2) and Eq (19), respectively (S1 Fig and S3 Fig).

#### 9. Experimental procedures

Data were collected from 1 adult male monkey (*macaca fascicularis*). Animal preparation and general methods were described previously [29]. In brief, anesthesia was induced with ketamine (10 mg/kg) and maintained during surgery with isoflurane (1.0–2.5% in 95% O2). During recordings, anesthesia was maintained by sufentanil citrate (6–18 μg/kg/hr, adjusted as needed). Vecuronium bromide (0.15 mg/kg/hr) was used to suppress eye movements. The use of anesthesia allowed us to present a large number of trials, while ensuring precise and reproducible retinal positioning across trials. All procedures were approved by the Albert Einstein College of Medicine at Yeshiva University and followed the guidelines in the United States Public Health Service Guide for the Care and Use of Laboratory Animals.

We recorded neuronal activity using arrays of 10 × 10 microelectrodes (400 μm spacing, 1 mm length) inserted in the opercular region of V1. Waveform segments that exceeded a threshold (a multiple of the RMS noise on each channel) were digitized (30 kHz) and sorted off-line. For all analysis we included signals from well-isolated single units as well as small multi-unit clusters, and refer to both as neurons.

We first measured the spatial RF of each neuron, using small gratings (0.5 degrees in diameter; 4 orientations; 250 ms presentation) presented at a range of positions. The receptive field center of each neuron was defined as the location of the peak of a 2-dimensional Gaussian fit to the spatial activity map (across the population, median *R* ^*2*^ = 0.79). We then measured the preferred orientation and spatial frequency of each neuron. Orientation tuning was measured with gratings drifting in 16 different directions, in 22.5 deg steps. Spatial frequency was measured at 4 orientations (0, 45, 90, and 135), with gratings whose spatial frequency varied between 0.1 and 8 cycles per degree. We used this information to align our stimuli with the center of the aggregate receptive field, and determine the orientation and spatial frequency of the stimuli closest to the preference of the sampled population.

The stimuli for the main experiment were static sinusoidal grating patches with a diameter of 2 degrees (100 pixels). The gratings’ Michelson contrast was 0.25 and the orientation was offset by {−7, 0, 14} degrees from the population preference. The pixel noise was drawn from a Gaussian distribution with standard deviation of either 0% (i.e., no noise) or 24% of the range of pixel values [0, 255]. To ensure that the spatial frequency content of the noise did not exceed the typical high frequency cutoff for parafoveal V1 (approximately 6 to 8 cycles/degree [30]), we first downsampled the gratings by a factor of 4 (corresponding to 12.5 pixels/degree), then added the pixel noise, and then upsampled by a factor of 4 by copying each pixel value in blocks of 4×4 pixels. After adding noise, pixels values outside the range [0, 255] were clipped.

We displayed stimuli on a calibrated CRT monitor (1024 × 768 pixels; 100 Hz frame rate; ~40 cd/m^2^ mean luminance) placed 110 cm from the animal, using custom software. All stimuli were displayed in pseudo-random order for 250 ms each, followed by a 250 ms uniform gray screen. Each stimulus was presented 900 times. Stimuli were presented monocularly in a circular aperture surrounded by a gray field of average luminance.

## Supporting Information

S1 Text Synthetic tuning curve model. Details of the model of synthetic tuning curves and covariances used for supporting S1 Fig and S3 Fig
(PDF)

S1 Fig (a) Histogram of differences between the predicted and empirical variance of the bias-corrected estimator, relative to the empirical variance. Simulations are based on the model described in S1 Text, with *N* = 100 neurons, 1000 simulated experiments and 200 trials per experiment per stimulus condition. The blue triangle at the top represents the mean relative difference. (**b**) Predicted variance (blue line) and empirical variance (dashed black line), as a function of the number of trials. The shaded area represents the standard deviation of the predicted variance across experiments.
(PDF)

S2 Fig Relative error for the decoder (a,b) and the bias-corrected estimator (c,d), for different population sizes: Continuous lines, *N* = 250, dashed lines, *N* = 50. Simulations are based on the model used in the main text, with the same parameters except the number of filters. (**a,c**) Fisher information in the original data. (**b,d**) Fisher information in the shuffled data. Note that in the shuffled data differential correlations are destroyed and the code is not robust, hence a slightly suboptimal decoder (e.g. one trained on finite data) is expected to miss much of the information, and to perform worse for larger than smaller populations. This is illustrated in (**b**), where the estimation error for the decoder increases with population size, as opposed to (**a**) where the error is relatively insensitive to population size. The direct estimator does not suffer from this issue: the estimation error for the shuffled information decreases, rather than increase, with population size (**d**).
(PDF)

S3 Fig (a) Each bar represents the relative error when using the direct estimator for the original data, the shuffled data, and the factorized decoder. Simulations are based on the model described in S1 Text, with *N* = 100 neurons, 1000 simulated experiments and 200 trials per experiment per stimulus condition. (**b-d**) Histograms of differences between the predicted and empirical Fisher information, for the original data (**b**), the shuffled data (**c**), and the factorized decoder (**d**). All histograms are centered at 0, hence the estimators are unbiased.
(PDF)

S4 Fig MSE for the data of Fig 6, for bias-corrected estimator (blue) and cross-validated decoder (red). The ground truth information value is not available for cortical data, therefore we used the arithmetic mean between the training set and validation set estimates obtained with the decoder at *T* = 900. Data are recorded from a population of *N* = 52 macaque V1 neurons. Conventions are as in Fig 6 in the main text.
(PDF)

S5 Fig Fisher information when removing correlations entirely by shuffling the data (top, denoted I_shuf_), and when decoding under the assumption that the data are independent (bottom, denoted I_diag_). Data are recorded from a population of *N* = 52 macaque V1 neurons. All conventions are as in Fig 5 in the main text.
(PDF)

S6 Fig Comparison of estimators at low spike counts. Data were generated using a model with Von Mises tuning curves and independent Poisson variability, with N = 50 neurons, and population-averaged spike count per trial matched to main Fig 7A and 7B. (**a**) Estimate of the Fisher information obtained by decoding (red) or direct estimation with bias correction (blue). The continuous lines represent the mean, the shaded area represents ±1 std across experiments, computed by bootstrap. (**b**) MSE of the decoder-based estimate (red) and the direct estimator (blue).
(PDF)

S7 Fig (a) Comparison between estimates obtained by the bias-corrected estimator (blue, BC) and different decoding methods. Left: early-stopping (CV), same as Fig 1B in main text; center: variational Bayes decoder (VB); leave-one-out cross validation with L2 regularization (LOOCV), with regularization parameter set to 0.1. Upper and lower bounds for the decoders correspond to information estimated from training set and validation set, respectively. Dashed black line denotes ground truth information. (**b**) Mean squared errors for all estimation methods. (**c**) Run time (in seconds) per experiment, for different estimators: Bias-corrected estimator (blue, BC); decoding with early-stopping (red, CV); variational Bayes decoder (brown, VB); leave-one-out cross validation with L2 regularization (green, LOOCV) with regularization parameter set to 0.1. Data were generated using the model of the main text, with *N* = 50 neurons. The code was run in Mathworks Matlab 7 (R2012a) on a workstation with Windows 7, processor Intel Core i7 2.70 GHz, 32 GB RAM.
(PDF)
