# Correction: Measuring Fisher Information Accurately in Correlated Neural Populations

**DOI:** 10.1371/journal.pcbi.1004945

**Published:** 2016-05-20

**Authors:** The PLOS Computational Biology Staff

There is an error in this paper. A factor of 1/2 has been omitted in the definition of a variable, in the following places in the paper:

- Results, first sentence: "theta^+ = theta + dtheta" and "theta^- = theta—dtheta" should be replaced by "theta^+ = theta + dtheta/2" and "theta^- = theta—dtheta/2"
- Methods section 1, second sentence, same correction as above.
- Methods section 2, second sentence, same correction as above.
- Methods section 3, second sentence, "theta +/- dtheta" should be replaced by "theta +/- dtheta/2"
- Equation 9, both appearances of "dtheta" should be replaced by "dtheta/2"
- Equation 12, "dtheta" should be replaced by "dtheta/2"

Please note that the factor is only missing in the definition of the variable, but it is correctly assumed in the equations that use that variable, as well as in the simulations, therefore this correction does not affect any of the results.

The authors thank Qianli Yang for pointing out the error.
