# Supplementary material for: Measuring Fisher Information Accurately in Correlated Neural Populations
Source: PLoS Comput Biol. 2015 Jun 1;11(6):e1004218. doi: 10.1371/journal.pcbi.1004218 (PMC4451760; doi:10.1371/journal.pcbi.1004218)
Supplement: S1 Text — Details of the model of synthetic tuning curves and covariances used for supporting S1 Fig and S3 Fig (PDF) [file pcbi.1004218.s001.pdf]

Text S1. Synthetic tuning curve model

Here we describe another model we used to simulate correlated population responses, following [12]. In this model, tuning curves are defined by Von Mises functions with random amplitudes a_i drawn from the log-normal distribution. Population responses are assumed Gaussian distributed, with standard deviations proportional to the tuning amplitudes (Poisson-like); the covariance between two neurons proportional to the product of the tuning amplitudes; and limited-range noise correlations (i.e. stronger correlations between neurons with similar stimulus preference):

$$\Sigma_{ij}(\theta) = \sigma_i(\theta)\sigma_j(\theta)\rho_{ij}$$

$$\sigma_i^2(\theta) = a_i f_i(\theta)$$

$$\rho_{ij} = c\left(\left|\theta_i \ominus \theta_j\right|\right) + \delta_{ij}\left(1 - c(0)\right)$$

$$c\left(\left|\theta_i \ominus \theta_j\right|\right) = c\left(0\right)\exp\left(2\cos\left(\left|\theta_i \ominus \theta_j\right|\right) - 2\right)$$

where $\ominus$ is the circular difference, and $c\left(0\right) = 0.2$. We used this model for S1 Fig. and S3 Fig. (see captions for simulation details) for the following reasons.

First, in Figure 5, we estimated I_{diag}. As explained in Results, a code where correlations limit information is redundant, meaning that little information loss results from using a slightly suboptimal decoder. In our simulations I_{diag} is very similar to the information extracted by the optimal decoder. We wanted to compare the two estimation methods (decoding and bias correction) in a case where I_{diag} is substantially smaller. Therefore in S3 Fig. we considered the model described here, in which noise correlations do not limit information (provided the tuning curves are heterogeneous).

Second, the ground-truth information computed for the main text model is approximate. S1 Fig. and S3 Fig. confirm that our predictions of the estimator bias and variance are correct, also for the model described here for which the ground truth information is known exactly.
