# Supplementary material for: Measuring Fisher Information Accurately in Correlated Neural Populations
Source: PLoS Comput Biol. 2015 Jun 1;11(6):e1004218. doi: 10.1371/journal.pcbi.1004218 (PMC4451760; doi:10.1371/journal.pcbi.1004218)
Supplement: S1 Fig — Simulations are based on the model described in S1 Text, with N = 100 neurons, 1000 simulated experiments and 200 trials per experiment per stimulus condition. The blue triangle at the top represents the mean relative difference. (b) Predicted variance (blue line) and empirical variance (dashed black line), as a function of the number of trials. The shaded area represents the standard deviation of the predicted variance across experiments. (PDF) [file pcbi.1004218.s002.pdf]

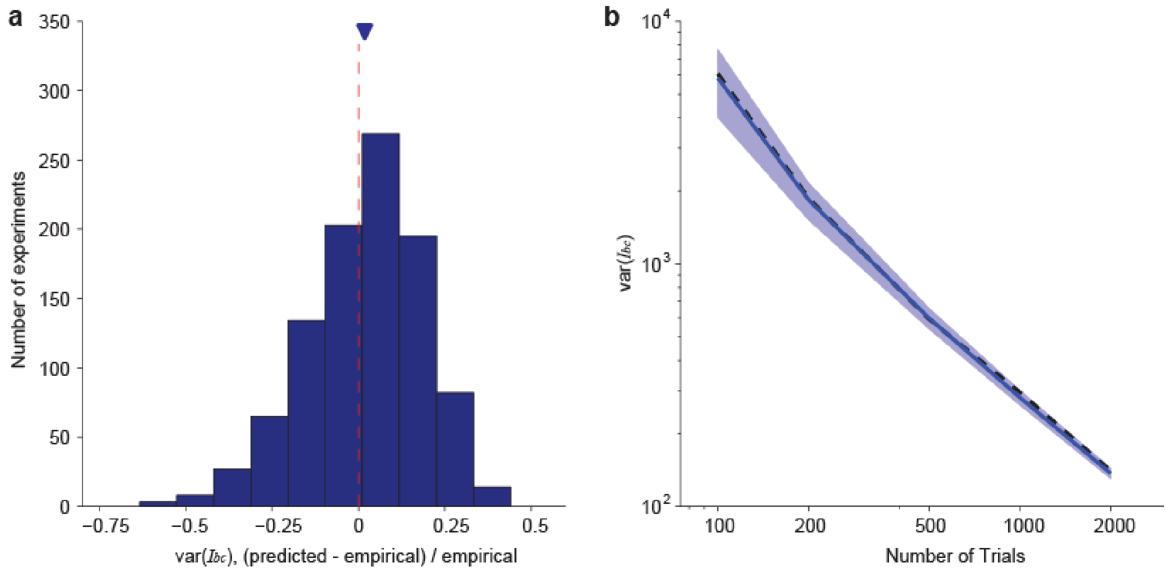

Figure S1. (**a**) Histogram of differences between the predicted and empirical variance of the bias-corrected estimator, relative to the empirical variance. Simulations are based on the model described in File S1, with *N*=100 neurons, 1000 simulated experiments and 200 trials per experiment per stimulus condition. The blue triangle at the top represents the mean relative difference. (**b**) Predicted variance (blue line) and empirical variance (dashed black line), as a function of the number of trials. The shaded area represents the standard deviation of the predicted variance across experiments.
