# Supplementary material for: Measuring Fisher Information Accurately in Correlated Neural Populations
Source: PLoS Comput Biol. 2015 Jun 1;11(6):e1004218. doi: 10.1371/journal.pcbi.1004218 (PMC4451760; doi:10.1371/journal.pcbi.1004218)
Supplement: S2 Fig — Simulations are based on the model used in the main text, with the same parameters except the number of filters. (a,c) Fisher information in the original data. (b,d) Fisher information in the shuffled data. Note that in the shuffled data differential correlations are destroyed and the code is not robust, hence a slightly suboptimal decoder (e.g. one trained on finite data) is expected to miss much of the information, and to perform worse for larger than smaller populations. This is illustrated in (b), where the estimation error for the decoder increases with population size, as opposed to (a) where the error is relatively insensitive to population size. The direct estimator does not suffer from this issue: the estimation error for the shuffled information decreases, rather than increase, with population size (d). (PDF) [file pcbi.1004218.s003.pdf]

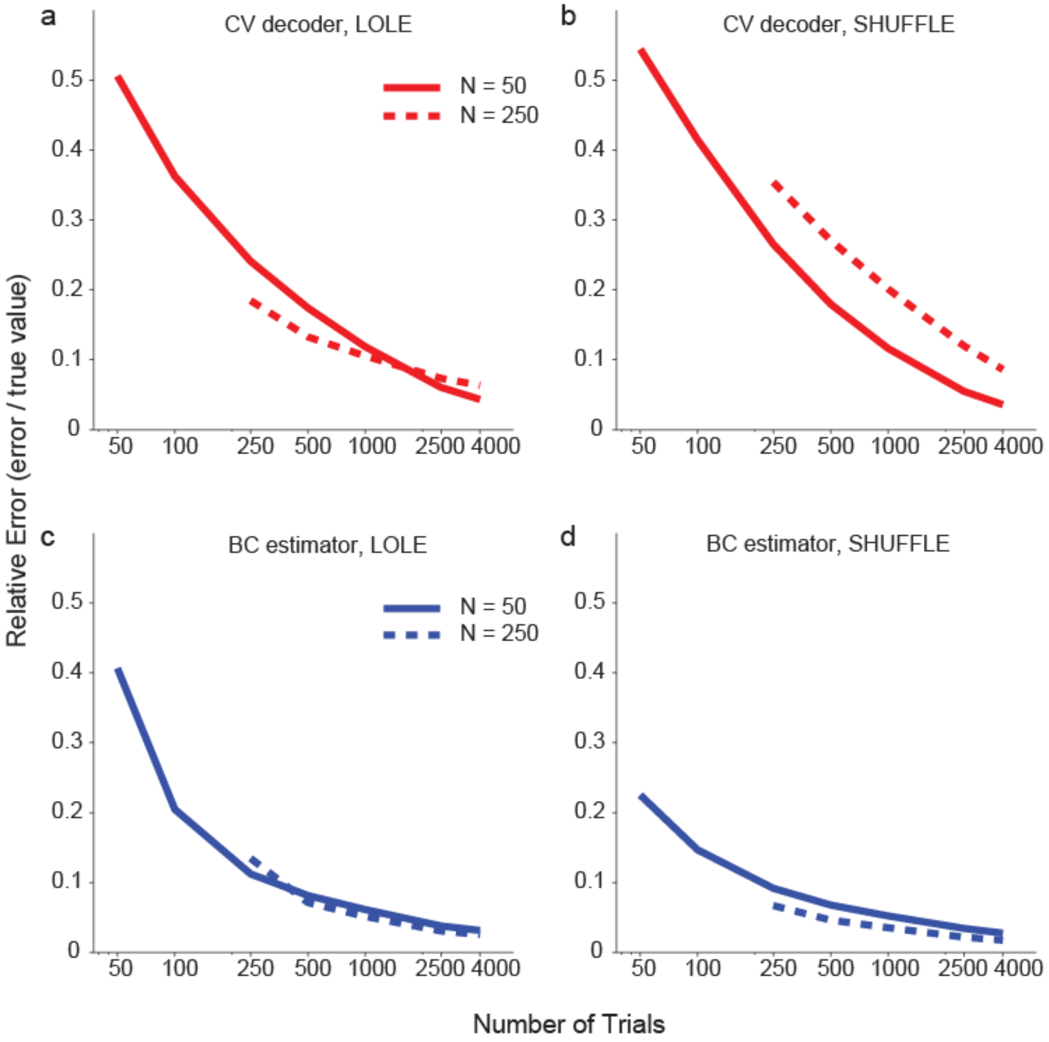

Figure S2. Relative error for the decoder (**a,b**) and the bias-corrected estimator (**c,d**), for different population sizes: Continuous lines, *N*=250, dashed lines, *N*=50. Simulations are based on the model used in the main text, with the same parameters except the number of filters. (**a,c**) Fisher information in the original data. (**b,d**) Fisher information in the shuffled data. Note that in the shuffled data differential correlations are destroyed and the code is not robust, hence a slightly suboptimal decoder (e.g. one trained on finite data) is expected to miss much of the information, and to perform worse as the size of the population increases. This is illustrated in (**b**), and can be contrasted to (**a**) where the error is relatively insensitive to population size. The direct estimator does not suffer from this issue: the estimation error for the shuffled information decreases, rather than increases, with population size (**d**).
