# Supplementary material for: Measuring Fisher Information Accurately in Correlated Neural Populations
Source: PLoS Comput Biol. 2015 Jun 1;11(6):e1004218. doi: 10.1371/journal.pcbi.1004218 (PMC4451760; doi:10.1371/journal.pcbi.1004218)
Supplement: S3 Fig — Simulations are based on the model described in S1 Text, with N = 100 neurons, 1000 simulated experiments and 200 trials per experiment per stimulus condition. (b-d) Histograms of differences between the predicted and empirical Fisher information, for the original data (b), the shuffled data (c), and the factorized decoder (d). All histograms are centered at 0, hence the estimators are unbiased. (PDF) [file pcbi.1004218.s004.pdf]

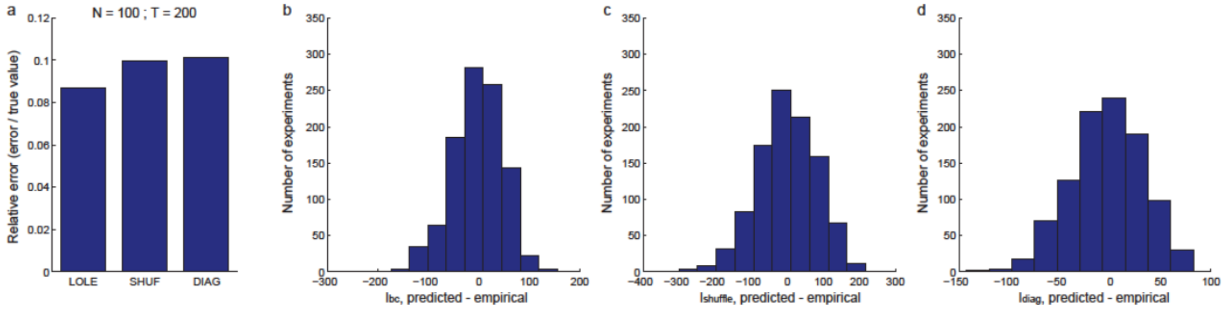

Figure S3. (**a**) Each bar represents the relative error when using the direct estimator for the original data, the shuffled data, and the factorized decoder. Simulations are based on the model described in File S1, with *N*=100 neurons, 1000 simulated experiments and 200 trials per experiment per stimulus condition. (**b-d**) Histograms of differences between the predicted and empirical Fisher information, for the original data (**b**), the shuffled data (**c**), and the factorized decoder (**d**). All histograms are centered at 0, hence the estimators are unbiased.
