# Supplementary material for: Measuring Fisher Information Accurately in Correlated Neural Populations
Source: PLoS Comput Biol. 2015 Jun 1;11(6):e1004218. doi: 10.1371/journal.pcbi.1004218 (PMC4451760; doi:10.1371/journal.pcbi.1004218)
Supplement: S4 Fig — The ground truth information value is not available for cortical data, therefore we used the arithmetic mean between the training set and validation set estimates obtained with the decoder at T = 900. Data are recorded from a population of N = 52 macaque V1 neurons. Conventions are as in Fig 6 in the main text. (PDF) [file pcbi.1004218.s005.pdf]

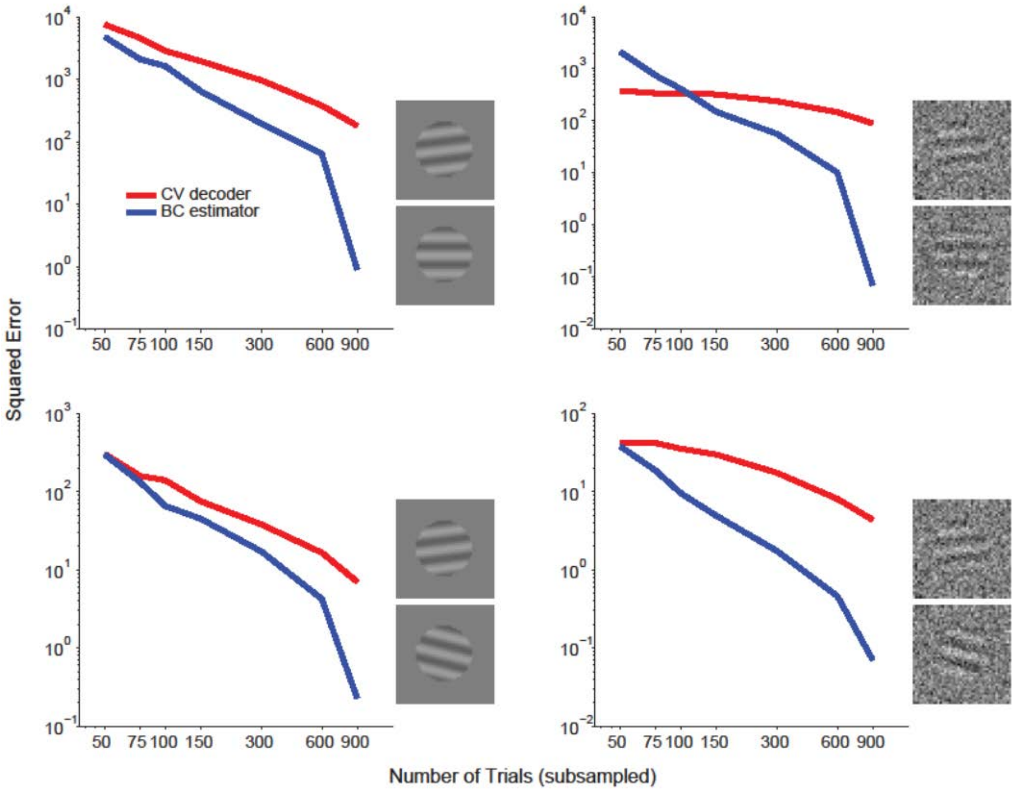

Figure S4. MSE for the data of Figure 6, for bias-corrected estimator (blue) and cross-validated decoder (red). The ground truth information value is not available for cortical data, therefore we used the arithmetic mean between the training set and validation set estimates obtained with the decoder at $T = 900$. Data are recorded from a population of *N*=52 macaque V1 neurons. Conventions are as in Figure 6 in the main text.
