# Supplementary material for: Measuring Fisher Information Accurately in Correlated Neural Populations
Source: PLoS Comput Biol. 2015 Jun 1;11(6):e1004218. doi: 10.1371/journal.pcbi.1004218 (PMC4451760; doi:10.1371/journal.pcbi.1004218)
Supplement: S5 Fig — Data are recorded from a population of N = 52 macaque V1 neurons. All conventions are as in Fig 5 in the main text. (PDF) [file pcbi.1004218.s006.pdf]

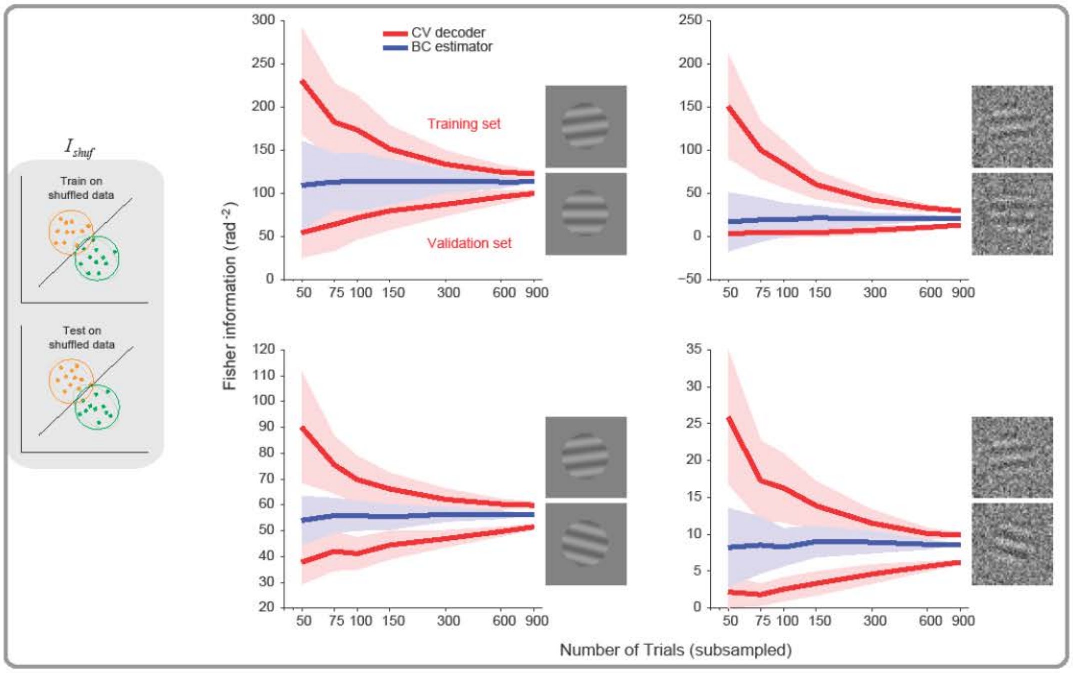

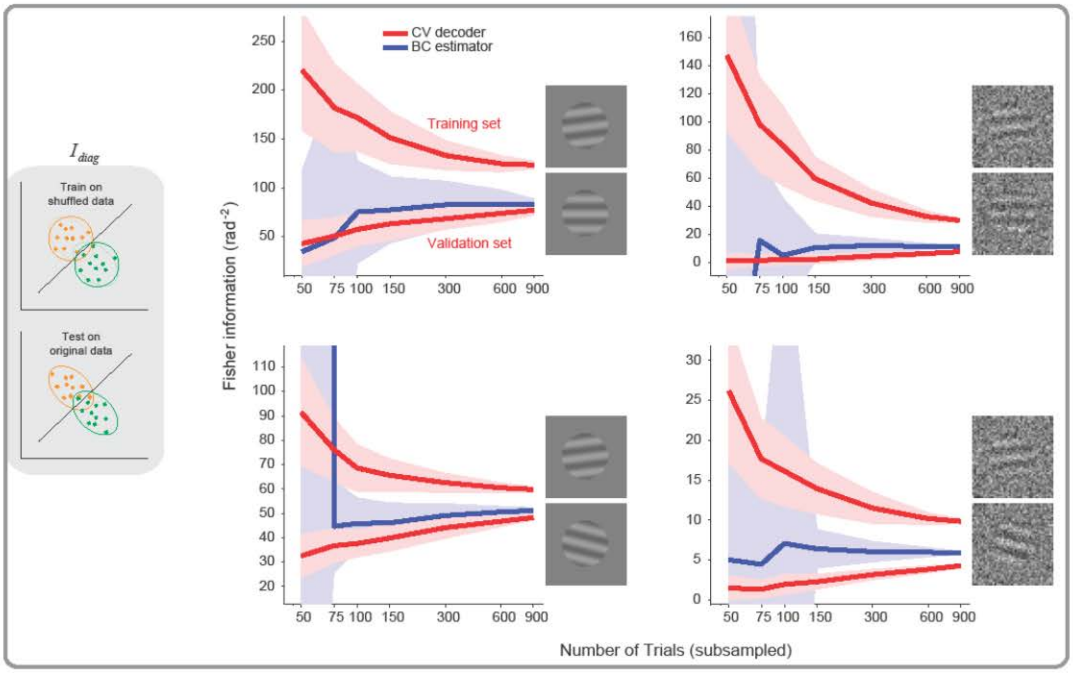

Figure S5. Fisher information when removing correlations entirely by shuffling the data (top, denoted I_{shuf}), and when decoding under the assumption that the data are independent (bottom, denoted I_{diag}). Data are recorded from a population of *N*=52 macaque V1 neurons. All conventions are as in Figure 5 in the main text.
