# Supplementary material for: Measuring Fisher Information Accurately in Correlated Neural Populations
Source: PLoS Comput Biol. 2015 Jun 1;11(6):e1004218. doi: 10.1371/journal.pcbi.1004218 (PMC4451760; doi:10.1371/journal.pcbi.1004218)
Supplement: S6 Fig — Data were generated using a model with Von Mises tuning curves and independent Poisson variability, with N = 50 neurons, and population-averaged spike count per trial matched to main Fig 7A and 7B. (a) Estimate of the Fisher information obtained by decoding (red) or direct estimation with bias correction (blue). The continuous lines represent the mean, the shaded area represents ±1 std across experiments, computed by bootstrap. (b) MSE of the decoder-based estimate (red) and the direct estimator (blue). (PDF) [file pcbi.1004218.s007.pdf]

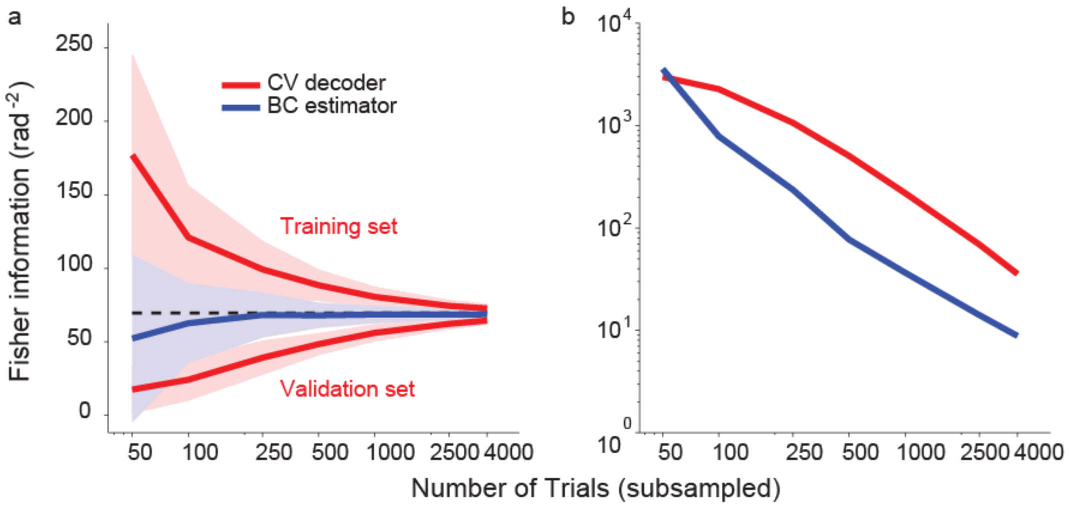

Figure S6. Comparison of estimators at low spike counts. Data were generated using a model with Von Mises tuning curves and independent Poisson variability, with N=50 neurons, and population-averaged spike count per trial matched to main Figure 7a,b. (**a**) Estimate of the Fisher information obtained by decoding (red) or direct estimation with bias correction (blue). The continuous lines represent the mean, the shaded area represents ±1 std across experiments, computed by bootstrap. (**b**) MSE of the decoder-based estimate (red) and the direct estimator (blue).
