# Supplementary material for: Measuring Fisher Information Accurately in Correlated Neural Populations
Source: PLoS Comput Biol. 2015 Jun 1;11(6):e1004218. doi: 10.1371/journal.pcbi.1004218 (PMC4451760; doi:10.1371/journal.pcbi.1004218)
Supplement: S7 Fig — Left: early-stopping (CV), same as Fig 1B in main text; center: variational Bayes decoder (VB); leave-one-out cross validation with L2 regularization (LOOCV), with regularization parameter set to 0.1. Upper and lower bounds for the decoders correspond to information estimated from training set and validation set, respectively. Dashed black line denotes ground truth information. (b) Mean squared errors for all estimation methods. (c) Run time (in seconds) per experiment, for different estimators: Bias-corrected estimator (blue, BC); decoding with early-stopping (red, CV); variational Bayes decoder (brown, VB); leave-one-out cross validation with L2 regularization (green, LOOCV) with regularization parameter set to 0.1. Data were generated using the model of the main text, with N = 50 neurons. The code was run in Mathworks Matlab 7 (R2012a) on a workstation with Windows 7, processor Intel Core i7 2.70 GHz, 32 GB RAM. (PDF) [file pcbi.1004218.s008.pdf]

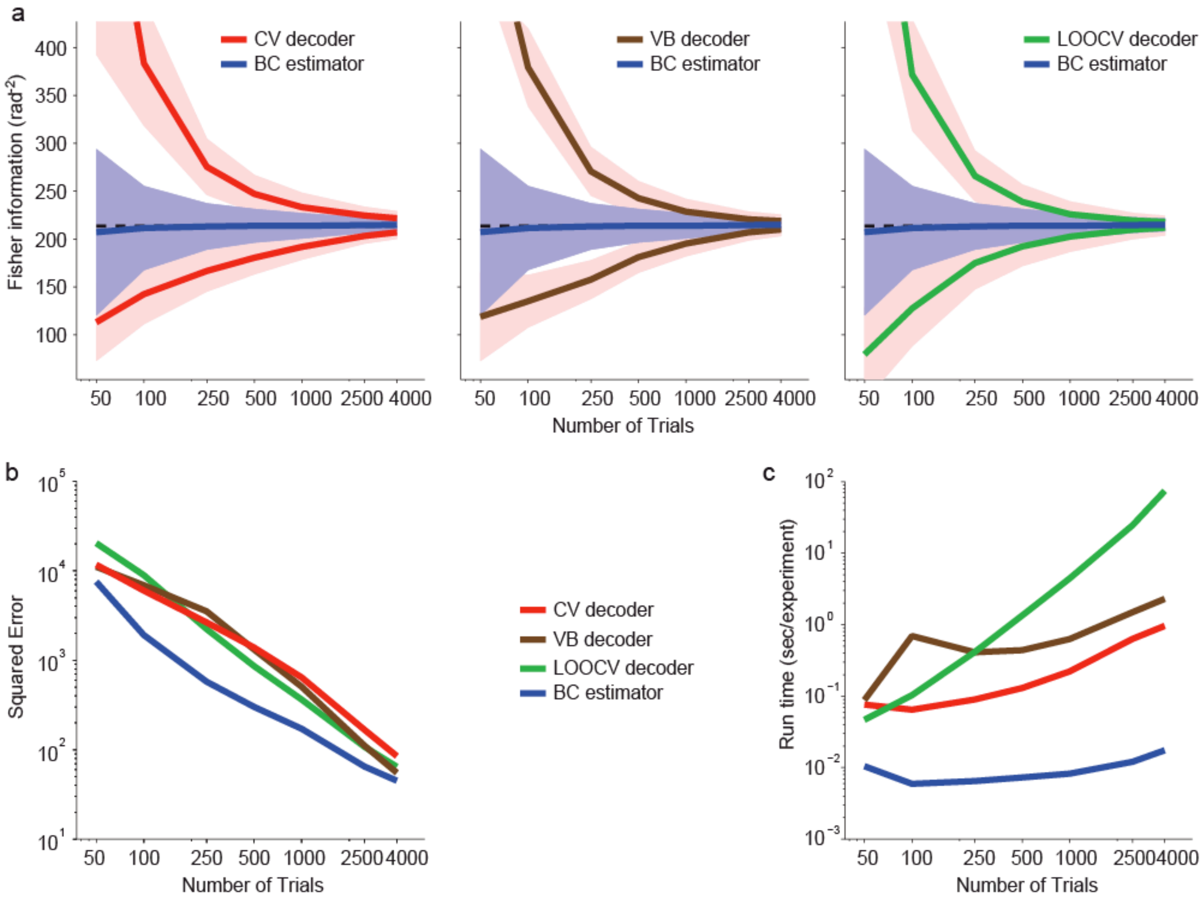

Figure S7. (**a**) Comparison between estimates obtained by the bias-corrected estimator (blue, BC) and different decoding methods. Left: early-stopping (CV), same as Figure 1b in main text; center: variational Bayes decoder (VB); leave-one-out cross validation with L2 regularization (LOOCV), with regularization parameter set to 0.1 (small values of this parameter are a common trade-off between ensuring that the covariance matrix is not ill-conditioned while adding little bias). Upper and lower bounds for the decoders correspond to information estimated from training set and validation set, respectively. Dashed black line denotes ground truth information. (**b**) Mean squared errors for all estimation methods. (**c**) Run time (in seconds) per experiment, for all estimation methods. Data were generated using the model of the main text, with N=50 neurons. The code was run in Mathworks Matlab 7 (R2012a) on a workstation with Windows 7, processor Intel Core i7 2.70 GHz, 32 GB RAM.
